# Safety Landscape of Therapeutic Nanozymes and Future Research Directions

**DOI:** 10.1002/advs.202407816

**Published:** 2024-10-24

**Authors:** Nikolaos Tagaras, Haihan Song, Shafaq Sahar, Weijun Tong, Zhengwei Mao, Tina Buerki‐Thurnherr

**Affiliations:** ^1^ Laboratory for Particles‐Biology Interactions Swiss Federal Laboratories for Materials Science and Technology (Empa) St. Gallen 9014 Switzerland; ^2^ Department of Health Sciences and Technology ETH Zurich Zurich 8093 Switzerland; ^3^ MOE Key Laboratory of Macromolecular Synthesis and Functionalization Department of Polymer Science and Engineering Zhejiang University 866 Yuhangtang Rd Hangzhou 310058 China; ^4^ College of Chemical and Biological Engineering MOE Key Laboratory of Macromolecular Synthesis and Functionalization Department of Polymer Science and Engineering Zhejiang University 866 Yuhangtang Rd Hangzhou 310058 China

**Keywords:** enzymatic activity, inflammation, oxidative stress, safety assessment, therapeutic nanozymes, toxicity

## Abstract

Oxidative stress and inflammation are at the root of a multitude of diseases. Treatment of these conditions is often necessary but current standard therapies to fight excessive reactive oxygen species (ROS) and inflammation are often ineffective or complicated by substantial safety concerns. Nanozymes are emerging nanomaterials with intrinsic enzyme‐like properties that hold great promise for effective cancer treatment, bacterial elimination, and anti‐inflammatory/anti‐oxidant therapy. While there is rapid progress in tailoring their catalytic activities as evidenced by the recent integration of single‐atom catalysts (SACs) to create next‐generation nanozymes with superior activity, selectivity, and stability, a better understanding and tuning of their safety profile is imperative for successful clinical translation. This review outlines the current applied safety assessment approaches and provides a comprehensive summary of the safety knowledge of therapeutic nanozymes. Overall, nanozymes so far show good in vitro and in vivo biocompatibility despite considerable differences in their composition and enzymatic activities. However, current safety investigations mostly cover a limited set of basic toxicological endpoints, which do not allow for a thorough and deep assessment. Ultimately, remaining research gaps that should be carefully addressed in future studies are highlighted, to optimize the safety profile of therapeutic nanozymes early in their pre‐clinical development.

## Graphical abstract

Nanozymes spark attention as emerging nanotherapeutics due to their impressive medicinal potential across a wide range of diseases. Nevertheless, beyond therapeutic efficacy, nanomedicines must undergo rigorous safety assessment to ensure successful clinical translation. This comprehensive review outlines the current safety landscape of therapeutic nanozymes and highlights future research directions aimed at enhancing the current safety assessment.

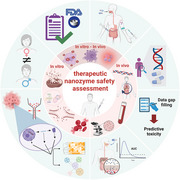

## 1 Introduction

Oxidative stress, a pathological condition, is the outcome of excessive ROS production caused by the intracellular imbalance between oxidant and antioxidant mechanisms. Generally, ROS are vital for redox signaling which has been shown to be among the driving forces of physiological functions, including but not limited to cell survival and proliferation.^[^ ^1^ ^]^ Representative examples of beneficial ROS functions in the human body include the enhancement of immune defence through oxidative burst and inflammasome activation, strengthening of inhibitory synaptic transmission in the central nervous system, and facilitation of spermatozoa maturity.^[^ ^2^, ^3^, ^4^, ^5^ ^]^ Nevertheless, disruption of the redox balance leads to an excess of harmful ROS which can react with biomolecules (e.g., DNA, proteins) to obstruct essential cellular functions. Hence, suppression of oxidative stress is of utmost importance to prevent disease development.

Inflammation, a tightly orchestrated process involving the activation of immune cells in response to endogenous or exogenous insults, is a vital protective mechanism to eliminate such insults and ensure the healing process. Inflammation can be divided into “sterile” or “non‐sterile” based upon its trigger. Non‐sterile inflammation responds to pathogen infections, through the recruitment of immune cells, such as neutrophils and macrophages that recognize pathogen‐associated molecular patterns (PAMPs).^[^ ^6^ ^]^ In contrast, sterile inflammation, a pathogen‐independent process, is activated by internal tissue or cellular damage and elicits the recognition of damage‐associated molecular patterns (DAMPs).^[^ ^6^, ^7^ ^]^ For a successful pathogen elimination or tissue repair, ROS are necessary, yet they can damage biomolecules, leading to chronic inflammation, instead of its resolution.^[^ ^6^ ^]^

Oxidative stress and inflammation processes are highly interconnected and interdependent.^[^ ^6^ ^]^ Consequently, they have been linked to the onset and persistence of a plethora of diseases, ranging from cardiovascular to neurodegenerative.^[^ ^8^, ^9^, ^10^ ^]^ Regrettably, to date, the therapeutic arsenal to combat inflammation lacks effective and safe approaches, regardless of the malignancy. For instance, preclinical successful drugs for the treatment of Alzheimer's disease (e.g., Verubecestat: BACE1 inhibitor) failed in the clinical phase due to poor efficacy and significant side effects.^[^ ^11^ ^]^ Equally, treatment of ischemic stroke relies on the FDA‐approved tissue plasminogen activator. However, this drug has a prominent risk of intracerebral hemorrhage.^[^ ^12^ ^]^ In addition, the scourge of multidrug resistant bacterial infections has driven antibiotics into a deadlock.^[^ ^13^ ^]^ In the case of cancer, the situation is even more complicated due to the tumor microenvironment (TME), which exhibits hypoxia, high glucose and glutathione (GSH) levels. Except surgical tumor removal, the current standard cancer treatment entails chemoradiotherapy, which has devastating side effects and its efficacy is hampered by the TME. The drawbacks of the current therapies and, at the same time, the global burden for mortality and morbidity such diseases account for, stimulate the urgent need for developing novel therapies.

**Figure 1:**
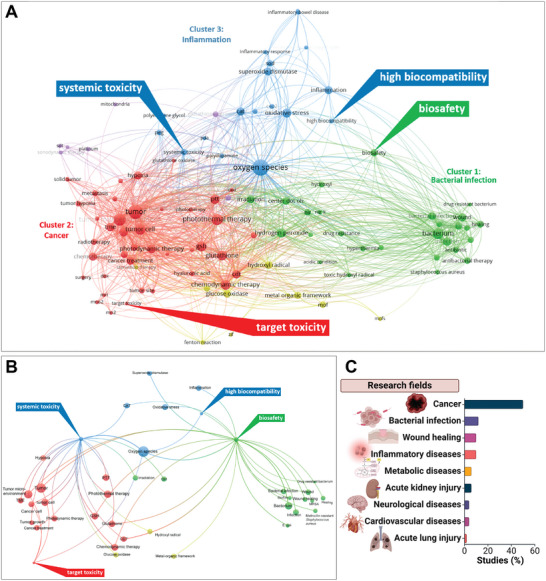
Graphical representation of key aspects of nanozyme research in the biomedical field. A) Bibliometric analysis (performed with VOSviewer) on the toxicity‐related 519 studies and identification of toxicity‐related keywords. Parameters used: titles and abstracts (including structured abstract labels), binary counting, minimum ten times occurrence, 60% cut‐off relevance, removal of general words (e.g., day, conclusion, activity), 1000 links were used to show the most robust connections. The color indicates the cluster, the sphere size indicates the importance, and the links indicate correlations. B) Reduced network showing only the interactions between toxicity‐related terms (i.e., systemic toxicity, target toxicity, high biocompatibility, and biosafety) and other biomedical terms. C) Exploitation of nanozymes in various medicine fields, based on the exemplary fraction of 50 studies used for the assessment of the current safety landscape.

Nanozymes, a ground breaking research area in the nanotechnology field, have garnered the attention of the scientific community as emerging nanotherapeutics due to their superior stability compared to the natural enzymes.^[^ ^14^ ^]^ The term “nanozyme” was coined in 2004, based on the striking similarity to catalytic polymers (synzymes), when it was discovered that functionalized Au nanoparticles (NPs) could catalyze transphosphorylation.^[^ ^15^ ^]^ Three years later Gao et al. discovered that Fe_3_O_4_ possess intrinsic peroxidase‐mimicking properties.^[^ ^16^ ^]^ Since then, the ongoing nanozyme research has expanded in various medicine fields (**Figure** **1**) and shows great potential toward redox‐mediated in vitro and in vivo therapeutic mechanisms.

Notably, since nanozymes are a new generation of enzyme‐like nanomaterials, besides the evaluation of their therapeutic potential, nanosafety assessment is a key aspect toward clinical application. Nanozymes stand out compared to conventional nanomaterials since they require additional attention for their biocompatibility. Nanotoxicity may stem from two aspects, namely their inherent physicochemical properties^[^ ^17^ ^]^ but also their enzymatic activity. Depending on the redox‐based treatment, nanozymes may exert oxidant or antioxidant enzymatic activity and it is crucial to exclude potential side effects in off‐target cells/tissues.

Given the superior advantages of nanozymes compared to their natural counterparts, the nanozyme‐biomedical application research has skyrocketed. There is already a great number of literature reviews concentrating on their properties, enzymatic activities, and ultimately their therapeutic potential,^[^ ^18^, ^19^, ^20^, ^21^, ^22^ ^]^ yet overlooking the safety aspect. Similarly, a vast number of primary research papers have been published, demonstrating their impressive medicinal capabilities in a broad range of diseases, however, only scratching the surface of their toxicity profile. In order to capture the rapid and expansive increase of nanozyme‐based therapy‐related studies, we screened the scientific literature database “Web of Science” using (“nanozyme” OR “nanozymes” AND “therapy”) as keywords. This search resulted in 1776 studies (as of September 2024 – articles and reviews) published between 2010 and 2024, with rapidly increasing numbers since 2018/2019, when the field started to evolve. To further extract the studies reporting data on the toxicity of therapeutic nanozymes, we included toxicity‐related keywords to the above‐mentioned ones, such as (“toxicity” OR “safety” OR “biocompatibility” OR “side effects”). This search reduced the number to 519 studies (as of September 2024 – articles and reviews), which gives the first indication that safety is often not a key parameter in the early design of therapeutic nanozymes. We performed a bibliometric analysis, (text‐mining functionality; VOSviewer software; version 1.6.20^[^ ^23^ ^]^), to construct and visualize co‐occurrence networks of important terms extracted from the collected literature on therapeutic nanozyme toxicity assessment (Figure 1A) and to identify dynamic and relationship patterns of toxicity‐related terms with other biomedical terms (Figure 1B). The generated science mapping portrays three well‐defined term clusters, namely infection‐related (e.g., bacterium, antibiotic), cancer‐related (e.g., tumor, chemotherapy), and inflammation‐related (e.g., inflammation, superoxide dismutase) clusters. Interestingly, the toxicity words have distinct relationships with biomedical terms (Figure 1B). For instance, “biosafety” belongs and has higher affinity to the infection‐related cluster, followed by inflammation‐related and cancer‐related clusters, pointing out that in the cancer therapy field the center of attention is more on the therapeutic efficacy than the safety of the drug, given the high severity and burden of the disease. In contrast, “systemic toxicity”, even though in the inflammation‐related cluster, is also highly linked to cancer‐related cluster, rendering it an important parameter in the development of nanozymes for cancer therapy. Finally, “target toxicity” is linked to the cancer‐related cluster, indicating perhaps a therapy‐ rather than a safety‐focused term. On this ground, our review aims to provide an overview of the current safety landscape of nanozymes from an exemplary fraction of 50 studies covering distinct medical application fields (Figure 1C) published between 2019 and 2024. Moreover, we identify and highlight pivotal gaps in the safety assessment of therapeutic nanozymes with the goal to form the foundations of supporting the safe development of these promising nanomaterials and accelerate their translation into the clinics.

## 2 Nanozymes: Overview and Characteristics

Nanozymes are defined as nanomaterials with enzyme‐like properties. They can mimic catalytic activities of natural enzymes including oxidoreductase [e.g., superoxide dismutase (SOD), peroxidase (POD), and nitrate reductase] as well as hydrolase (e.g., esterase, nuclease, and silicatein) activity.^[^ ^24^ ^]^ Importantly, nanozymes have unique advantages over their natural counterparts related to higher stability, lower cost production, versatile engineering of catalytic function, and performance under a broader range of conditions. Since, in this review, our main theme and focus is the safety aspect of nanozymes, we will give only a brief overview about nanozymes, their types, and synthesis methods for our readers to understand the basics of this emerging new field and its potential. For our readers who are more interested in understanding the basics of this field and would like to dive deeper, including detailed synthesis, characterization, mechanisms, and diverse types of nanozymes we recommend the following referenced literature.^[^ ^25^, ^26^, ^27^, ^28^, ^29^ ^]^

### 2.1 Classical Nanozymes: Advantages and Disadvantages Compared to Natural Enzymes

Enzymatic catalysis is an inextricable part of life's activities, guaranteeing that thousands of reactions in the body are carried out methodically and at high speed under mild conditions.^[^ ^30^, ^31^, ^32^ ^]^ Given the fundamental role of enzymes in the maintenance of homeostasis and physiology of living organisms, scientists have focused on engineering nanomaterial‐based enzyme mimics with improved stability, efficiency, and specificity, in order to overcome the drawbacks of natural enzymes such as the complexity of the purification process, easy inactivation under harsh conditions, and high costs.^[^ ^33^, ^34^, ^35^, ^36^ ^]^

The discovery in 2004 and 2007 that Au and Fe_3_O_4_ NPs can exert a catalytic/enzyme‐like activity was a milestone, triggering scientists to dive deeper and to define nanomaterials with natural enzyme‐like activities as “nanozymes”.^[^ ^18^ ^]^ Consequently, they started summarizing their conformational relationship and the key influencing factors of their activities^[^ ^19^ ^]^ in order to drive the next‐generation of artificial enzymes.^[^ ^37^ ^]^ With the emergence of nanotechnology, nanozymes have been gradually enriched with a variety of materials, including but not limited to metal oxides,^[^ ^38^, ^39^, ^40^, ^41^, ^42^ ^]^ noble metals,^[^ ^43^, ^44^, ^45^, ^46^ ^]^ carbon materials,^[^ ^47^, ^48^, ^49^, ^50^ ^]^ polymers^[^ ^51^, ^52^, ^53^ ^]^ and metal‐organic frameworks (MOFs).^[^ ^54^, ^55^, ^56^ ^]^ Composite nanozymes, combining the advantages of each component,^[^ ^57^, ^58^, ^59^ ^]^ and single‐atom nanozymes with atomically distributed active centers^[^ ^60^, ^61^, ^62^, ^63^ ^]^ are nowadays at the forefront of this research field, allowing their activities to mimic a great variety of oxidoreductases, as well as hydrolases and isomerases.^[^ ^20^, ^64^, ^65^ ^]^

As a non‐biological molecule, the biggest nanozyme advantage compared to natural enzymes, is the superior stability and maintenance of structure and properties in extreme conditions.^[^ ^21^, ^66^ ^]^ Other advantages cover the simplicity of preparation and functionalization, low cost, and long‐term storage.^[^ ^20^, ^64^, ^67^ ^]^ However, natural enzymes are composed of complex and fine spatial structures, allowing the formation of reaction sites with specific substrate recognition, endowing them with unique selectivity. In contrast, nanozymes due to the lack of substrate recognition, catalyze a variety of substrates indiscriminately, rendering nanozyme catalysis non‐selective.^[^ ^22^, ^68^ ^]^ To address this issue, Zhang's group used doping to introduce single atoms into gold clusters.^[^ ^69^ ^]^ Similarly, Li's group used iron doping to modulate the electron energy of the carbon dot (CD) nanozyme conduction band, thereby matching/mismatching the energy barriers of the enzymatic reaction. In addition, it allowed the manipulation of the electron transfer between the nanozyme and the substrate by forming an electron lock and thus steering the selectivity of the nanozymes.^[^ ^70^ ^]^ It is believed that with the gradual exploration of scientists, the selective catalysis of nanozymes will eventually be realized. Furthermore, nanozymes lack a special structure found in natural enzymes called “channel”. The channel connects the surface of the enzyme to its catalytically active center, which allows the transportation of the substrate, ensuring an efficient enzyme catalysis.^[^ ^71^ ^]^ Nanozymes lack such structures, resulting in surface‐mediated catalysis and consequent lower activity efficiency. These differences lead to certain difficulties in studying the catalytic mechanism of nanozymes.^[^ ^72^, ^73^ ^]^

### 2.2 Emerging Single Atom Nanozymes (SAzymes)

The structural and morphological resemblance of classical nanozymes remains controversial as they do not mimic natural enzymes, which have single catalytically active center while the rest of the protein structure assists in binding the substrates and transferring them to the catalytically active centers.^[^ ^60^, ^74^, ^75^ ^]^ When the materials are scaled down to the atomic size, their properties change entirely compared to their bulk form due to the change in energy, which expresses itself in terms of a change in quantum effects. These changes in quantum effects eventually have a drastic impact on the electronic properties, optical properties, strength, and magnetism of the nanozymes.^[^ ^76^, ^77^ ^]^ Besides these fascinating effects, the reduction in size imparts these materials with exponential increase in the surface area, providing high substrate‐catalyst binding site interaction on the interfaces of the catalyst thus improving the catalytic, adsorption, and interaction properties.^[^ ^78^, ^79^ ^]^ Moreover, in bulk form, the nanozyme activities are difficult to tune and the selectivity of the substrates is not ensured as there are multiple catalytically active sites. This may lead to complications such as toxicity or other unwanted and unpredictable catalytic interferences when these nanozymes are used in biological tissues or other biomedical applications (e.g., biosensing).^[^ ^80^ ^]^

Controlling the size of these nanozymes, especially downscaling them to nanoclusters or single atoms to obtain specific and precise catalytic activities, has attracted massive attention from the scientific community. With a reduced size, the surface atoms on the nanozyme increase exponentially, along with variations in the surface defects, electronic structure, and atomic structure, which greatly enhances the utilization efficiency of the metal and improves catalytic activities remarkably.^[^ ^81^ ^]^ Zhang and co‐workers in 2011 coined the term “single‐atom catalysts” (SACs) for their work on single platinum atoms anchored on FeOx NPs that possessed exceptionally high CO oxidation activity. Therefore, SACs could be defined as those nanomaterials that have active isolated sites in the form of single atoms stabilized on a support or are alloyed with other metal NPs.^[^ ^81^ ^]^ SACs are considered as the limit of precision at the atomic level that one can achieve in the design of nanomaterials. SACs possess excellent catalytic efficiencies and selectiveness due to the homogenous distribution of their active sites on the support's surface with maximum atom‐utilization efficiency and depict easily predictable geometric structure.^[^ ^82^ ^]^ The metal‐support interactions in SACs enable enhanced charge‐transfer effects within the whole structure and a low‐coordination environment, which results in fully exposed active sites and thus better intrinsic activity of the active single metal sites. As these materials lack the presence of metal‐metal bonds, their coordination structure resembles closely to the natural enzymes due to the presence of M‐N_4_ coordination such as that observed in metal‐porphyrin ring, featuring the geometric, chemical, and electronic configuration and arrangement of metalloenzymes. For instance, proximal ligand of horseradish peroxidase (HRP), cytochrome P450 and oxymyoglobin enzymes have a single heme b cofactor as a catalytically active site, similar to Fe‐N_4_ configuration in Fe‐based SACs. Hence, in recent years, numerous studies have explored the potential of SACs in mimicking natural enzymes and their biomedical applications.

**Figure 2:**
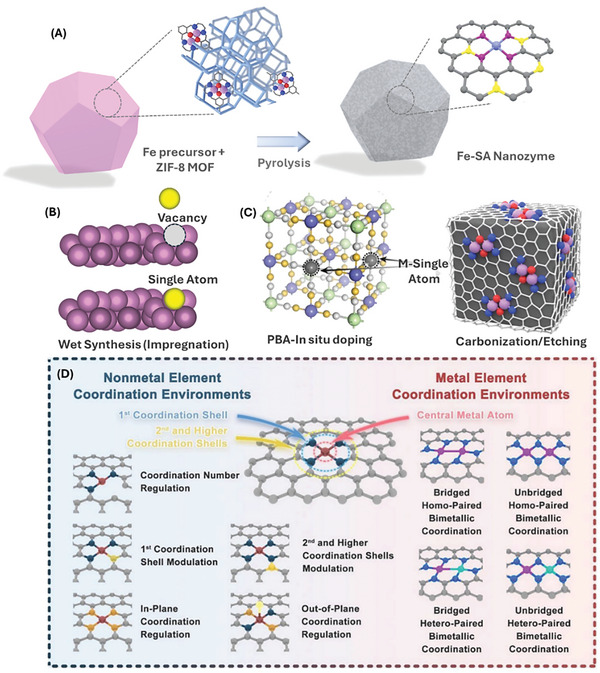
Synthesis strategies and type of supports for SAzymes including A) MOF‐based support via pyrolysis, B) metal‐based supports via impregnation, C) in situ PBA doping, and D) altering various coordination environment strategies in carbon‐based matrix. Reproduced with permission.^[^ ^83^ ^]^ Copyright 2023, Wiley.

During the synthesis of SACs, it is ensured that there is no metal‐metal bond formation. This is achieved through backing the metal single atoms on some kind of support. Generally, the supports used for the doping of single atoms include metal oxides, carbons, zeolites, MOFs, and covalent organic frameworks (COFs). The single metal atoms implanted in the host metal surface, stimulate different kinds of charge transfer activities and alter the cohesive energy, resulting in the tuning of the catalytic performance.^[^ ^84^ ^]^ Various metal supports such Ni, Au, Cu, Al, and Fe substrates have been used to incorporate Pt single atoms while some studies have also reported the loading of Pd single atoms on Cu, Ag, and Au substrates (**Figure** **2**B).^[^ ^85^, ^86^, ^87^, ^88^, ^89^, ^90^ ^]^ In metal oxides supports, the single‐atom anchoring strategy takes place through the creation of defects by introducing oxygen or metal atom vacancies, resulting in the formation of single‐atom alloys (SAA). In Fe_2_O_3_ support, the ability of the Fe sites to reduce themselves is utilized to anchor another single metal atom, while the presence of oxygen vacancies in CoOx, MnOx, TiO_2_, and CeO_2_ have been employed to embed the single atoms in these supports.^[^ ^91^, ^92^, ^93^, ^94^, ^95^ ^]^ A variety of carbonaceous materials including g‐C_3_N_4_ derivatives, CDs defect‐containing graphene, carbon nanotubes (CNTs), biomaterial‐ and polymer‐derived carbons have been used as support for single atoms.^[^ ^96^, ^97^, ^98^, ^99^, ^100^, ^101^ ^]^ The coordination environment in first shell has the most impact on activity of a nanozyme as a result of direct covalent bonding between carbon ligand atoms and single metal atom.^[^ ^102^, ^103^ ^]^ However, the second and higher shell coordination environment also significantly influences the electronic structure of single metal active sites and changes the nanozyme activity or efficiency.^[^ ^102^, ^103^ ^]^ Heteroatoms in the carbon framework are necessary to achieve strong metal atom–support interactions. As the sp2 carbon lacks lone pair electrons, it cannot interact with the metal atom due to the minimal binding energy. This results in very weak metal‐support interactions leading to higher surface diffusional mobility of single metal atoms with a tendency to agglomerate and form metal clusters. Hence, the defect‐free graphite and graphene surfaces are unsuccessful for the support of SACs. To solve this problem, the introduction of other heteroatoms such as N, O, P, and S as coordination atoms for the effective binding of single atoms on the carbon support are generally introduced in the carbon framework.^[^ ^25^ ^]^ Nitrogen, as coordination atom for single metal atom binding, is considered an ideal heteroatom, having higher electronegativity, which would allow stronger coordination with the metal and a beneficial modification in the electronic structure of carbon, thus leading to better catalytic performance.^[^ ^104^, ^105^, ^106^ ^]^ The general coordination type utilized in most studies include the MX_4_ site where the single metal atom is coordinated with four heteroatoms, thus forming a porphyrin‐like structure with square‐planar geometries. Recently, many studies have also explored the MX_2_, MX_3_, MX_5_ (M: metal, X: N, O, S, P halogen) coordination through tuning of the number of heteroatoms attached on the coordination sites (Figure 2D).^[^ ^107^ ^]^ In this aspect, density functional theory (DFT) plays a crucial role in determining and predicting the structures and models of heteroatom incorporation in the carbon framework, as well as the single atom attachment and coordination in the framework. Porous materials, including MOFs and COFs, are also considered an ideal support for the anchorage of single metal atoms as they offer very high surface areas that allow better adsorption of substrates on SACs. Various MOF supports such as Zn‐based bimetallic zeolite imidazole framework (ZIF‐8), UiO‐66, Al‐based porphyrinic MOF (AlOH)‐_2_H_2_TCPP, MOF‐525, MOF‐808, and PCN‐222 have been reported in literature as supports for single‐atom synthesis.^[^ ^108^, ^109^, ^110^, ^111^, ^112^, ^113^ ^]^ More recently, COFs have also been explored as support for single atoms as they have exposed functionalities that could easily coordinate and support the single metal atoms. COF‐1 utilizing the precursors 4,4′‐(benzo[c][1,2,5]thiadiazole‐4,7‐diyl)dianiline (Bt–NH_2_) and 5′‐(porphyrin‐5,15‐diyl) diisophthalaldehyde (P–CHO) was utilized as a source to generate COF‐X. As a result, COF2, COF3, and COF4 were yielded through the alteration of the amine monomer with Pz–NH_2_, TP–NH_2_, and DMTP–NH_2_, used to load Au single atoms.^[^ ^114^ ^]^ Other COFs studied for loading single atoms include [Re‐(bpy)‐(CO)3Cl], DQTP, TAPB‐BPDA COF‐367, and bipyridyl‐based COF (COFbpy‐M).^[^ ^114^, ^115^, ^116^, ^117^, ^118^ ^]^

### 2.3 Synthesis Approaches of SAzymes

SAC engineering with fixed coordination structure and high proportion of catalytically active sites or single atom loading is particularly essential to their biomedical applications. The fabrication of SACs, however, is confronted with multiple challenges as there is a very high tendency of atomic aggregation and metal clusters formation during the synthesis procedure of SACs and post‐treatment.^[^ ^119^ ^]^ To address this issue, some major approaches for highly precise SAC synthesis have been proposed including, among others, wet‐chemistry strategies, pyrolysis, atomic layer deposition (ALD).^[^ ^120^ ^]^

Generally, the wet‐chemistry approach to design SACs involves a series of consecutive steps, including i) attaching the precursor with metal‐based single‐atom species on the support through coprecipitation, impregnation, electrostatic absorption or ion‐exchange, ii) calcination or drying at high temperatures to purge the unwanted ligands and iii) activation or reduction processes.^[^ ^121^ ^]^ Various synthesis approaches reported in literature for SACs on different supports are given in **Table** **1**. However, the metal atom species may lean toward aggregation and formation of metal nanoclusters during the synthetic procedure. To meet the standards of biomedical applications, highly accessible single‐atom active sites are necessary. Reinforcement of the interaction between the support and metal atoms is critical in suppressing the generation of metal clusters. Highly active ligands with greater electronegativity, including N, O, and S functional groups, display better interactions with single‐metal atom species employed for engineering the coordination sites and avoiding cluster formation of metal atoms.^[^ ^25^ ^]^ Even though SAC fabrication using wet chemistry approaches may show great potential in the biomedical field, there are numerous concerns that are required to be tackled in order to achieve successful therapeutic applications. For instance, when SACs are administered through intravenous (IV) injection for in vivo applications, their blood circulation and distribution in the body rely significantly on their surface characteristics and hydrodynamic diameter.^[^ ^22^ ^]^ Other critical issues include toxicity, stability, and leaching due to coordination with other biological entities in vivo.^[^ ^122^ ^]^ Any adverse effects induced by SACs should be comprehensively assessed to guarantee their desirable biosafety for in vivo use.

**Table 1:** Types of metal supports used for single‐atom anchorage and their synthesis routes.

Support	Nanozyme	Single atom	Activity	Synthesis approach	Ref
Metal oxides/ sulfides	Pd/CeO_2_	Pd	SOD CAT POD	Aqueous phase synthesis (hydrothermal, 95 °C for 6 h).	[151]
	Pd–CeO_2_@GOx	Pd	CAT POD GSH‐Px	Pd sites anchored on CeO_2_ nanosheets were synthesized first using salt precursor pyrolysis at 650 °C for 2 h in air. Pd single atoms loaded by impregnation in solution and then high‐temperature calcination at 200 °C for 2 h under 5% H_2_/Ar. GOx was loaded through electrostatic interactions. Assembly leaching method by mixing Co precursor salt and MoS_2_ nanosheets through ultrasonication at 4 °C for 24 h.	[152]
	SA Co–MoS_2_	Co	POD	Assembly leaching method by mixing Co precursor salt and MoS_2_ nanosheets through ultrasonication at 4 °C for 24 h.	[153]
	Cu‐CeO_2_	Cu	POD	Coprecipitation and hydrothermal synthesis (180 °C for 6 h).	[154]
	Ag/CeO_2_	Ag	POD OXD	CeO_2_ synthesis through hydrothermal route at 40 °C using a reducing agent. Next, coprecipitation of Ag under reducing conditions via hydrothermal method at 120 °C for 5 h.	[155]
	Pd@CeO_2_/N‐PC‐rGO	Pd	SOD CAT POD OXD	Coprecipitation method under reducing environment using N_2_ at 70 °C to obtain Pd@CeO_2_. N‐PC‐rGO was obtained via a pyrolysis procedure of ZIF‐8‐GO. 60 min of stirring at 70 °C of the above two precursors to obtain Pd@CeO_2_/N‐PC‐rGO.	[156]
Carbons	2D‐Cu–N–C	Cu	OXD	Template‐assisted synthesis/ coordination/ pyrolysis (750 °C for 2 h under N_2_) and etching.	[157]
	FeSA@CQDs‐1 & FeSA@CQDs‐2	Fe (III) Fe (II)	POD SOD CAT POD	EDTA complexation with Fe(II) and Fe(III) salts, low temperature pyrolysis (350 °C in N_2_ at ramp rate of 2 °C/min. Ultrasonication and dialysis to obtain single‐atom CDs.	[100]
	Cu–N–C	Cu	POD	Salt template‐assisted synthesis, pyrolysis (Ar atmosphere at 750 °C (5° C/min) for 2 h.	[158]
	FeN_4_P_2_	Fe	POD	Secondary atom‐assisted strategy by adding phytic acid through complex template method to form P–Fe–polypyrrole nanowires. Pyrolysis in N_2_/NH_3_ 900 °C for 30 min followed by a step of acid washing.	[159]
	FeN_4_C–SOx	Fe	POD	Slurry mixture of L‐cysteine and melamine and FeCl_3_ as source of S, C, and Fe. Two‐stage pyrolysis in Ar (first stage: from 20 °C to 550 °C, for 2 h and then heated to 800 °C for 2 h) ramp rate 2 °C/min.	[160]
	Co_1_/C_3_N_4_	Co	NAD^+^/NADGH regeneration	Co precursor, dicyanamide (DCD), and crystallite template were mixed together and freeze‐dried to form Co‐DCD @NaCl precursor. Then pyrolyzed at 550 °C in N_2_ for 4 h, followed by removal of NaCl crystals.	[161]
	ZnBNC	Zn	POD	Coordination of salt and 2‐methylimidazole (2‐MI) with addition of boric acid and pyrolysis 1000 °C for 3 h in Ar.	[162]
	Ce‐N‐C		Phosphatase	Coprecipitation of CTAB‐APS polymer and Ce salt precursor and vigorous stirring for 24 h, followed by pyrolysis at 900 °C under N_2_ for 30 min and NH_3_ for 30 min followed by acid etching.	[163]
MOFs	FeN_5_ SA/CNF	Fe	OXD	Host‐guest assisted MOF structure, pyrolysis (900 °C under N_2_).	[164]
	MnSA‐N_3_‐C	Mn	POD OXD GSH OXD	Host‐guest assisted MOF assembly (Mn‐ZIF‐8), pyrolysis 900 °C.	[107]
	MoSA–Nx–C	Mo	POD OXD	MoO_2_(acac)_2_ encapsulation in ZIF‐8 and pyrolysis at 800, 900 and 1000 °C under Ar to obtain Mo–N_4_–C, Mo–N_3_–C, and Mo–N_2_–C.	[165]
	pFeSAN	Fe	OXD	Hemoglobin@ZIF‐8 was pyrolyzed at 900° C under Ar to decompose hemoglobin and remove Zn^2+^.	[60]
	Co/PMCS	Co	SOD CAT GPx	Pyrolysis of Co‐doped ZIF‐8 (900 °C under N_2_) 3 h.	[108]
	PtTS	Pt	POD	Pt clusters were obtained through reduction and attachment on ZIF‐8 followed by pyrolysis and atomization to break Pt‐Pt bonds at 1050 °C for 5 h (ramp rate of 5 °C/min) in N_2_.	[166]
	OxgeMCC‐r SAE	Ru	CAT	Collective coordination, hydrophobic, and electrostatic interactions among organic linker [Co(C≡N)_6_], PVP polymer, photosensitizer, and metal ions.	[167]

Pyrolysis approach for the synthesis of SACs is predominantly employed for the fabrication of carbon‐based supports.^[^ ^123^ ^]^ Generally, the pyrolysis approach involves high‐temperature calcination (∼500–1000 °C), removal of residual metal species and metal clusters through acid leaching using H_2_SO_4_ or other strong acids, followed by thermal post‐treatment, essential in restoring the destruct carbon structure.^[^ ^124^ ^]^ The atomically‐scattered metal nodes in MOFs and coordination‐driven carbon‐based SACs have a highly tuned coordination environment, assisting in the rational tailoring of the steric configuration of SACs, due to the tunable structures of carbons and MOFs (Figure 2A,D). The metal nodes especially in MOFs and Prussian blue analogues (PBAs) could thus be transformed in situ into single isolated metal atom sites distributed on the carbon support with N doping via the organic ligand carbonization (Figure 2C).^[^ ^125^, ^126^ ^]^ For instance, typical SAC incorporation on MOF support reported in literature utilizes the 1,2‐methylimidazole ligand coordinated with Zn^2+^ ions to form ZIF‐8.^[^ ^127^ ^]^ The salt precursor of the required metal atom to be doped on the MOF is mixed with ZIF‐8 solution and the precipitate is pyrolyzed at various temperatures from 800–1200 °C, resulting in the evaporation of Zn atoms and generation of free N sites in ZIF‐8, allowing the coordination with other desired single metal atoms.^[^ ^128^, ^129^, ^130^ ^]^ Similarly, the two metals separated by C and N in PBAs are employed for the generation of single‐atom sites. The metal‐metal cores generated as a result of pyrolysis are etched with acid to form graphene heteroatom‐doped SACs.^[^ ^125^, ^126^, ^131^ ^]^ This strategy ensures a very high single atom loading efficiency (19.5%), which is necessary for better catalytic performance of a nanozyme.^[^ ^126^ ^]^ In case of carbons, like graphene, the surface is etched and treated with various strong oxidizing or reducing agents. This leads to the formation of defects in the graphene framework and attachment of various functionalities such as ‐COOH, NH_2_, and –OH, which are essential in holding the single atoms.^[^ ^132^, ^133^, ^134^ ^]^ Graphene sometimes is also treated and functionalized through high temperature pyrolysis, where the organic or inorganic precursor source of heteroatoms is placed upstream in a process called chemical vapor deposition (CVD). The carbon precursor in the tube furnace and the gas flow along the column lead to evaporation of heteroatoms and their doping in the carbon structure, utilized for the coordination of single‐metal atoms through post‐thermal treatment.^[^ ^135^, ^136^, ^137^ ^]^ The SACs synthesized using pyrolysis may confront complications in homogeneous solution as they tend to have high surface energies, leading to nanozyme agglomeration. To ensure their homogenous dispersibility in biological environments, it is essential to modify them with biofriendly surfactants/polymers that could enhance their dispersibility, enabling in vivo biomedical applications.

Atomic layer deposition (ALD) comprises of consecutive self‐limiting reactions between the gaseous precursors and substrate, offering a controllable approach to realize homogeneous anchorage of single‐metal atoms on the porous supports with high surface areas. ALD could assist the coordination of single‐metal atoms in a self‐terminated pattern through exposing the chosen precursor support to the pulsing vapors of the precursor metal atom to be doped.^[^ ^138^ ^]^ This strategy ensures maximal precision for the synthesis of precise SACs, and it is speculated that it will encompass other approaches for the synthesis of SACs in the future. It is worth‐mentioning that ALD is a little different from CVD, a similar chemical vapor‐phase deposition procedure, which utilizes a continuous stream of precursor and is distinguished by non‐self‐limiting growth.^[^ ^139^ ^]^ An umbrella term known as energy‐enhanced ALD, (EE‐ALD) is generally used to cover a variety of procedures that use energy transfer to metal atom precursors before they bind to their substrates or supports. EE‐ALD includes hot wire ALD, plasma‐enhanced ALD (PE‐ALD), and ALD approaches using ozone gas.^[^ ^140^ ^]^ Ozone can be produced either through O_2_ plasma or by using UV light radiation. EE‐ALD practices are generally employed to lower the deposition temperature, to avoid the water consumption as the source of oxygen precursor, or to enhance the growth rates.^[^ ^141^ ^]^ ALD offers high precision in developing SACs, however, its limitations such as high operation costs and low yield are hindering the further advancement in SAC fabrication for biomedical applications.

Characterization techniques play a crucial role in identifying and understanding SACs. Such techniques differ from the ones of bulk nanomaterials due to the precision technology required to function accurately at an atomic level. In situ/operando techniques are employed to study the catalyst and providing insights into its behavior under reaction conditions. These techniques include X‐ray photoelectron spectroscopy (XPS), X‐ray Absorption Spectroscopy (XAS), and Extended X‐ray Absorption Fine Structure (EXAFS) which are utilized for probing the chemical and compositional properties of catalyst surfaces, providing information on the local structure.^[^ ^142^, ^143^, ^144^, ^145^, ^146^ ^]^ Electron microscopy, including transmission electron microscopy (TEM) and scanning transmission electron microscopy (STEM), can visualize individual atoms on the nanozyme surface.^[^ ^147^ ^]^ High‐Resolution Transmission Electron Microscopy (HRTEM), Aberration‐corrected high‐angle annular dark‐field scanning transmission electron microscopy (AC‐HAADF‐STEM), and Scanning Tunnelling Microscopy (STM) provide detailed visualization of single metal atoms on catalyst surfaces with atomic resolution.^[^ ^148^, ^149^, ^150^ ^]^ These techniques collectively provide a comprehensive understanding of the structure, composition, and behavior of SACs, helping in the design and optimization of catalysts.

## 3 In Vitro Safety Landscape of Nanozymes

**Figure 3:**
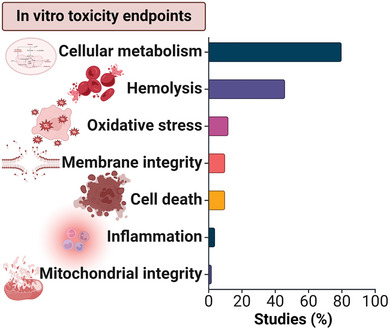
In vitro toxicological endpoints addressed in scientific studies evaluating the therapeutic potential of nanozymes.

Safety screening in the drug discovery process starts with an in vitro toxicity assessment that includes an array of in vitro tests and has the benefit of predicting early safety liabilities, before a drug is promoted for further in vivo assessment. The in vitro toxicity endpoints, reported in the present cohort of studies include cellular metabolism, membrane integrity, hemolysis, oxidative stress, cell death, inflammatory responses, and mitochondrial activity (**Figure** **3**). In the following section, we will briefly summarize the approaches and key findings on nanozyme toxicity from in vitro (**Table** **2**) hazard assessment investigations according to individual toxicity endpoints.

**Table 2:** Studies conducting in vitro toxicity assessment of therapeutic nanozymes. Normal font: no toxic effect, italic font: mild to moderate effect.

Material	Nanozyme Therapy	Size [nm]	Activity	Cell type	Viability assay/ concentration/ exposure time/ outcome	Hemolysis study: erythrocyte origin/ concentration/ exposure time/ hemolytic rate	Oxidative stress assay/ concentration/ exposure time/ outcome	Other endpoints: assay/ concentration/ exposure time/ outcome	Ref.
Metals	Au_1_Pd_3_‐FA Cancer	2‐3	SOD MPO	AML12	CCK‐8/ 0.3‐0.6 mm/ 24 h/ No viability decrease	NA	NA	NA	[46]
	Ag@Pt‐NIL/HA Liver fibrosis	60‐70	SOD CAT	LX‐2 L02	*CCK‐8/ 6.25‐100 mg mL^−1^/ 24 h/ LO‐2: No viability decrease, LX‐2: 100 µg mL^−1^: ∼20% viability decrease*	Mouse/ 6.25‐100 mg mL^−1^/ 2 h/ <2%	NA	NA	[191]
	Cu_2‐x_Se‐PEG‐GOx Cancer	110.1‐127.5	CAT GOx POD GSH OXD	L929	MTT/ 50–200 µg mL^−1^/ 24 h/ No viability decrease	NA	NA	NA	[192]
	Pd@Pt‐T790 Bacterial infection	50	CAT	HUVEC NIH‐3T3	MTT/ 12.5‐100 ppm/ 24 h/ No viability decrease	Mouse/ 20–200 ppm/ 3 h/ <2%	NA	NA	[193]
	Zr^4+^‐Ru^3+^/Pt^4+^‐Ce6@HA Cancer	125	CAT POD GSH OXD	HUVEC	CCK‐8/ 10–400 µg mL^−1^/ 24 h/ No viability decrease	NA	NA	NA	[194]
	Se@SiO_2_–Mn@Au/DOX Cancer	120	GOx POD	IEC‐18	CCK‐8/ 25–400 µg mL^−1^/ 24 h/ No viability decrease	ND/ 25–400 µg mL^−1^/ 4 h/ <4%	NA	NA	[195]
	Fe@melittin pro‐peptide Cancer	≈124	POD OXD GSH OXD	L929	*CCK‐8/ 25–200 µg mL^−1^/ 24 h/ 200 µg mL^−1^: ≈25% viability decrease*	ND/ 50–400 µg mL^−1^/ 4 h/ <4%	NA	NA	[196]
Metal oxides	DEX‐CeO_2_ Urinary tract infection	2‐3	SOD CAT RNS scavenger	RAW264.7 T24	CCK‐8/ 100–500 µg mL^−1^/ 24 h/ No viability decrease	NA	NA	NA	[41]
	PAA‐Fe_3_O_4_@GOx Skin injury	289.2	GOx POD	HEK‐293	CCK‐8/ 50–250 µg mL^−1^/ 24 h/ No viability decrease	ND/ 50–250 µg mL^−1^/ 4 h/ <5%	NA	Cell death: Annexin V/ 100, 250 µg mL^−1^/ 24 h/ Negligible apoptosis	[178]
	RuO_2_ Kidney injury	≈2	SOD CAT POD GPx	HEK293	Calcein‐AM/PI/ MTT/ 25 µg mL^−1^/ 24 h/ No viability decrease	NA	H_2_DCF‐DA/ 20 µg mL^−1^/ 24 h/ No ROS generation Peroxy Orange/ 20 µg mL^−1^/ 24 h/ No H_2_O_2_ increase	*Cell death*: Annexin V, PI/ 20 µg mL^−1^/ 24 h/ NPs: 14.6%, control: 6.04% *Mitochondrial integrity*: JC‐1/ 20 µg mL^−1^/ 24 h/ MMP: no significant change	[181]
	NCeO_2_‐PEI‐MoS_2_ Cancer	200‐350	SOD CAT	HBL‐100	MTT/ 6–200 µg mL^−1^/ 24 h/ No viability decrease	NA	H2DCF‐DA/ 0.25‐1 mg mL^−1^/ 24 h/ No ROS generation	n.a.	[197]
	MS‐CeO_2_‐miR 129 Skin injury	60‐100	CAT	HaCaT	CCK‐8/ 10–30 µg mL^−1^/ ND/ No viability decrease	NA	NA	NA	[198]
	Au‐DNA‐Fe_3_O_4_ Cancer	712	GOx POD	HEK‐293	MTT/ 5–200 ppm/ 24 h/ No viability decrease	NA	H_2_DCF‐DA/ 100 µg mL^−1^/ 24 h/ No ROS generation	NA	[199]
	OA‐MnO_2_ Bacterial infection + Skin injury	50	POD OXD	HUVEC	*MTT/ 0.01‐0.8 mm/ 24 h/ ≥ 0.01 mm: ≈20% viability decrease*	NA	NA	NA	[200]
	Fe_3_O_4_/Ag/Bi_2_MoO_6_ Cancer	35	SOD CAT POD GSH OXD	HaCaT	MTT/ 6.25‐150 µg mL^−1^/ 24 h/ No viability decrease	ND/ 25–150 µg mL^−1^/ ND/ <5%	NA	NA	[201]
	MoS_2_‐PEI‐CeO_2_ Cancer	≈415	POD GSH OXD	HCT116	*Alamar blue/ 5–200 µg mL^−1^, PEI M_w_: 0.2‐25 kDa/ 24 h/ ≥100 µg mL^−1^: dose‐dependent viability decrease (100 µg mL^−1^: ∼20% decrease). PEI‐dependent viability decrease*	Human/ 5–200 µg mL^−1^/ 24 h/ <6%	NA	NA	[202]
	SNLP‐IrO_x_‐BS12 Bacterial infection	37	POD	NIH‐3T3 HUVEC	*MTT/ 1.95‐250 µg mL^−1^/ 24 h/ 250 µg mL^−1^: ≈20% viability decrease*	Mouse/ 15.625‐250 µg mL^−1^/ 4 h/ <3%	NA	NA	[203]
	Fe_3_O_4_‑GOx Diabetic ulcer	13	CAT POD GOx	HUVEC	MTT/ 5–200 µg mL^−1^/ 24 h/ No viability decrease	NA	NA	NA	[204]
	CeO_2_‐PA Cancer	ND	SOD CAT	LX‐2 HepG2/ADR	Alamar Blue/ 1–1000 µg mL^−1^/ 24 h/ No viability decrease	NA	H_2_DCF‐DA/ 50 µg mL^−1^/ 6 h/ No ROS generation	NA	[205]
	Cu_x_O@EM‐K Alzheimer's	≈70	SOD CAT GPx	HL‐7702	MTT/ 25–400 µg mL^−1^/ 24 h/ No viability decrease	Mouse/ 25–400 µg mL^−1^/ 3 h/ <4%	NA	NA	[206]
	IR780‐MnO_2_‐PLGA Cancer	166	HPOD GSH OXD	Bend.3	CCK‐8/ 20–100 µg mL^−1^/ 24 h/ No viability decrease	NA	NA	NA	[207]
	Citrate‐Mn_3_O_4_ Huntington's	≈6	POD GPx	HEK‐293	*MTT/ 2.5‐800 µg mL^−1^/ 24 h/ 200 µg mL^−1^: ∼20% viability decrease*	NA	NA	NA	[208]
	Cu_2_MoS_4_ Bacterial infection	≈28	POD OXD	HEK‐293 HeLa	LDH/ 5–80 µg mL^−1^/ 24 h/ No viability decrease Calcein AM/PI/ 5–80 µg mL^−1^/ 24 h/ HeLa: No viability decrease, HEK‐293: not tested	*Mouse/ 5–80 µg mL^−1^/ 3 h/ <10%*	NA	NA	[209]
	CeO Acute inflammation	2.8	SOD CAT	HepG2 Renca SVEC4‐10 RAW264.7	Live/dead staining/ 0.1‐2.5 mg mL^−1^/ 24 h/ No viability decrease	NA	NA	NA	[210]
	MnOx‐coated Au Cancer	≈150	NADPH OXD POD	L929	*MTT/ 25–400 µg mL^−1^/ 24 h/ ≥25 µg mL^−1^: dose‐dependent viability decrease (25 µg mL^−1^: ∼25% decrease)*	ND/ 50–400 µg mL^−1^/ ND/ <2%	NA	NA	[211]
Carbons	N/C Cancer	ND	SOD NADH OXD GDH	HUVEC	*MTT/ 25–100 µg mL^−1^/ 24 h/ 100 µg mL^−1^: ≈20% viability decrease*	NA	NA	NA	[212]
	CDs Lung injury	2.7±0.7	SOD RONS scavenger	A549 RAW264.7 EA.hy926	*CCK‐8/ 0.1‐0.5 mg mL^−1^/ 24 h/ RAW264.7: ≤0.25 mg mL^−1^: viability increase, A549, EA.hy926: No viability decrease*	ND/ 6.25‐200 µg mL^−1^/ 3 h/ <2%	NA	NA	[213]
	CDs Inflammatory bowel disease	3	SOD ^●^OH scavenger	ND	NA	ND/ 10–3000 µg mL^−1^/ ND/ <10%	NA	NA	[214]
	V_2_N MXene Bacterial infection	200	POD OXD	ND	NA	*Mouse/ 5–100 µg mL^−1^/ 3 h/ <10%*	NA	NA	[215]
SAzymes	**MOF‐based**								
	Prussian Blue Skin injury	34±8	SOD CAT POD	NIH‐3T3	MTT/ 10–200 µg mL^−1^/ 24 h/ No viability decrease	NA	NA	Inflammation: RT‐qPCR, ELISA/ 50 µg mL^−1^/ 24 h/ TNF‐α (mRNA, protein) IL‐1β (mRNA): no changes	[184]
	Ca/Fe Prussian Blue Pancreatitis	≈7.5	SOD POD GPx RONS scavenger	AR42J	CCK‐8/ 25–1600 µg mL^−1^/ 24 h/ No viability decrease	Human/ ND/ 4 h/ ND (only qualitative images)	NA	n.a.	[216]
	PEG‐MOF/PtAu Cancer	ND	CAT OXD	COS7	*MTT/ 10–30 µg mL^−1^/ 24 h/ ≥20 µg mL^−1^ at normoxia with laser: ∼25% viability decrease. No viability decrease in hypoxia*	ND/ 5–500 µg mL^−1^/ 6 h/ 5–100 µg mL^−1^: ≈9–15%, 200, 500 µg mL^−1^: ≥ 30%	NA	NA	[217]
**Other**								
	PEG‐Cu‐HCF Cancer	102.5±21.8	POD GSH OXD	HEK‐293 L02	*MTT/ 12.5‐200 ppm/ 24 h, 48 h/ 24 h: No viability decrease, 48 h: 200 ppm: ≈35%, (HEK‐239) and ∼41% (LO2) viability decrease*	ND/ 20–1000 ppm/ ND/ <2%	H_2_DCF‐DA/ ND/ 12 h/ slight enhancement H_2_O_2_ detection/ ND/ 12 h/ No H_2_O_2_ change GSH/ ND/ 6 h/ No GSH change	Cell death: TUNEL/ ND/ 24 h/ L02: No apoptosis	[169]
	PVP‐Ir Kidney injury	1‐2	SOD CAT POD RNS scavenger	HEK‐293T	MTT/ 12.5‐400 µg mL^−1^/ 24, 48 h/ No viability decrease	Mouse/ 62.5–2000 µg mL^−1^/ 6 h/ ≤1%	NA	Cell death: Annexin V, PI/ 100 µg mL^−1^/ ND/ Negligible apoptosis	[179]
	Cu@MoS_2_‐PEG Cancer	ND	POD	L929	*CCK‐8/ 25‐100 µg mL^−1^/ 24 h/ 100 µg mL^−1^: ∼25% viability decrease*	NA	H_2_DCF‐DA/ 100 µg mL^−1^/ 24 h/ No ROS generation	Cell death: Calcein AM, PI/ 24 h/ Negligible apoptosis	[180]
	Co‐Fe_3_O_4_ Ischemic Stroke	45	CAT POD RNS scavenger	HT22	CCK‐8/ 6.25‐50 µg mL^−1^/ 24 h/ No viability decrease	NA	NA	Inflammation: ELISA/ 25 µg mL^−1^/ 24 h/ IL‐1β, IL‐6, TNF‐α: no significant effects	[183]
	B‐SA_50_ Inflammatory bowel disease	168	SOD CAT ^●^OH scavenger	HT29	MTT/ 0.625‐10 µg mL^−1^/ 24 h/ No viability decrease	NA	NA	NA	[218]
	Pt/CeO_2_ Cancer	≈1.6	SOD CAT POD OXD	L02	LDH, CCK‐8/ 25–250 µg mL^−1^/ 48 h/ No viability decrease	NA	NA	NA	[219]
	DSPE‐PEG_2000_‐RosA‐Mn Kidney injury	142	SOD CAT RONS scavenger	HK‐2	MTT, CCK‐8/ 0.5‐5 µg mL^−1^/ 24 h/ No viability decrease	Mouse/ 15.625, 31.25, 62.5, 125, 250 µg mL^−1^/ 4 h/ <1%	NA	NA	[220]
	Pt‐PCN224‐GOx‐EM Cancer	156.9±0.9	CAT GOx	HUVEC	CCK‐8/ 50, 100 µg/ 4, 8, 24 h/ No viability decrease	ND/ 50, 100 µg/ 0, 4, 8, 24 h/ <5%	NA	NA	[221]
	PtCu_3_‐PEG Cancer	14	HRPOD GPx	HUVEC	MTT/ 6.25‐100 µg mL^−1^/ 24 h/ No viability decrease	NA	NA	NA	[222]
	BSA‐Cu Cancer	7.6±0.9	POD GSH OXD	NCM460 HK‐2	CCK‐8/ 7.5‐60 µg mL^−1^/ 24 h/ No viability decrease	NA	NA	NA	[223]
	DOX‐Fe@ZIF‐8 Cancer	230	CAT POD	HepG2	Calcein AM/PI/ 125–500 µg mL^−1^/ 24 h/ No viability decrease	ND/ 125–500 µg mL^−1^/ ND/ <5%	NA	NA	[224]
	BSA@CeO/Fe^2+^ Cancer	3.46	SOD CAT POD	HUVEC L02	CCK‐8/ 0.5‐400 µg mL^−1^/ 24 h/ No viability decrease	NA	NA	NA	[225]
	SA‐Ce‐N_4_‐C‐OH_2_ Hyperglycemia	ND	SOD CAT POD OXD	HepG2	*CCK‐8/ 0.78‐50 µm/ ND/ ≥6.26 µm: dose‐dependent viability decrease (6.26 µm: ≈20% decrease)*	NA	NA	NA	[226]
	Cu‐DCA Diabetic skin injury	20–25	SOD CAT	HUVEC	CCK‐8/ 2.4‐24 µm of Cu/ 24 h/ No viability decrease	NA	NA	NA	[227]
	Fe@HA@GOx@PCN‐224 Cancer	170	POD GOx	ND	NA	Rabbit/ 12.5‐200 µg mL^−1^/ ND/ 1%	NA	NA	[228]
Smart assembly‐Nano ‐hybrid	Arg‐Gly‐Asp‐GOx‐CAT Cancer	65	CAT GOx	L02	*MTT/ 1–10 µg mL^−1^/ 24 h/ ≥1 µg mL^−1^: dose‐dependent viability decrease (1 µg mL^−1^: ≈20% decrease)*	Rat/ 10–200 µg mL^−1^/ 3 h/ <2%	NA	NA	[229]

Abbreviations: Nanozyme activities: SOD: Superoxide dismutase, CAT: Catalase, GOx: Glucose oxidase, OXD: Oxidase, GSH OXD: Glutathione oxidase, GPx: Glutathione peroxidase, POD: Peroxidase, HPOD: Hydrogen peroxidase, HRPOD: Horseradish peroxidase, MPO: Myeloperoxidase, GDH: Glucose dehydrogenase, RNS: Reactive nitrogen species, RONS: Reactive oxygen and nitrogen species. ND: Not determined, NA: Not assessed.

### 3.1 Cell Viability

Cell viability is a critical endpoint for hazard assessments as it provides information about cell survival and proliferation upon exposure to a foreign stimulus allowing the establishment of a dose‐response curve able to identify safe therapeutic concentrations. Cell viability assays mostly measure cellular metabolic activity or cell membrane integrity and can potentially give insights about the two main types of cell death, apoptosis and necrosis.^[^ ^168^ ^]^ According to Table 2, the majority of studies have used assays that measure the metabolic activity (e.g., MTT, CCK‐8) after 24 h of nanozyme exposure. Despite the large variety of nanozymes with highly distinct composition and physicochemical properties, many NPs did not elicit considerable acute toxicity in different cell types. However, cell viability was moderately decreased upon exposure to the following nanozymes: N/C, OA‐MnO_2,_ PEG‐Cu‐HCF, MoS_2_‐PEI‐CeO_2_, SNLP‐IrO_x_‐BS12, Citrate‐Mn_3_O_4_, Arg‐Gly‐Asp‐GOx‐CAT, SA‐Ce‐N_4_‐C‐(OH)_2_, Fe SA@melittin pro‐peptide, Ag@Pt‐NIL/HA, PEG‐MOF/PtAu, MnOx‐coated Au and Cu@MoS_2_‐PEG. In one study using PEG‐Cu‐HCF nanozymes,^[^ ^169^ ^]^ toxicity was not observed after 24 h, but was detected 48 h post‐treatment indicating that viability studies should include prolonged exposure times in particular if the nanozymes are accumulating and persisting in the cells. Importantly, the viability assessment of nanozymes was mostly evaluated in non‐cancerous cell lines representing the healthy tissue/organ fraction of the human body (generally fibroblasts and endothelial cells) to help understand potential off‐target toxicity. However, it would be important to also study toxicity in inflamed tissue since inflammation can increase susceptibility of cells to NPs.^[^ ^170^ ^]^

### 3.2 Hemocompatibility

The hemocompatibility of nanomedicines is a major concern since blood is the first contact point in the case of IV administration but also the responsible route for the distribution of NPs to tissues and organs. As soon as nanomedicines enter the bloodstream, they interact with different blood components (e.g., plasma proteins and blood cells), and thus interfere with their physiological function, posing a threat to normal body physiology. Different studies have demonstrated that NPs can hamper the coagulating system, leading to NP‐induced coagulopathy and perturbation of hemostatic balance.^[^ ^171^ ^]^ Generally, the investigation on NP‐blood impact is more thorough in vivo, since it evaluates the levels of various components such as hemoglobin, hematocrit, white blood cells, and so forth in a physiological organism. In contrast, in vitro hemocompatibility of nanomedicines is mainly investigated with specific assays for hemolysis, complement activation, platelet activation/aggregation, and coagulation.^[^ ^172^ ^]^ Among these endpoints, hemolysis can be used as an early and accurate biomarker of the toxic potential of nanomedicines, in addition to acting as a predictive tool for in vivo extrapolation.^[^ ^172^ ^]^ Our literature search of therapeutic nanozymes showed that most studies examined the hemolytic rate of erythrocytes (Table 2). According to a review published by the European Commission Joint Research Center (EU JRC), the criterion for the classification of a nanomaterial to be considered hemolytic is the cut‐off hemolysis percentage, namely non‐hemolytic (0–2%), slightly hemolytic (2–5%) and hemolytic (>5%).^[^ ^172^ ^]^ These hemolytic criteria are based on the ASTM (American Society for Testing and Materials) E2524‐22 standard, which is the standard test method for analysis of hemolytic properties of NPs. According to ASTM, the standard incubation period of NPs with blood is 3 h ± 15 min. From the pool of studies, only 5 out of 23 studies adopted the 3‐hour incubation period. Three of them can be considered slightly or non‐hemolytic and two hemolytic. From the rest of the 18 studies, seven did not reveal any hemolysis (<5%) even after longer incubation periods (4–24 h), five did not disclose the incubation time, two showed hemolysis but after 6 h and 24 h of incubation, one did not show hemolysis but only after 2 h of incubation, and three were highly inconclusive due to various reasons (i.e., no disclosure of nanozyme concentration and hemolytic rate, or showing absorbance results without translating them into a rate). Overall, several NPs have shown hemolytic activity and therefore, this endpoint should always be included in the hazard assessment of novel therapeutic nanozymes.

### 3.3 Oxidative Stress

Pathophysiological levels of ROS generation and, eventually, oxidative stress can be triggered by endogenous and exogenous triggers. Endogenous triggers encompass, among others, dysregulation of the mitochondrial electron transport chain or transmembrane NADPH oxidases.^[^ ^173^ ^]^ In contrast, exogenous triggers of intracellular ROS induction consist of the “exposome” which includes but is not limited to factors such as pollutants, toxicants, or NPs. As a result, oxidative stress is an important toxicological endpoint when performing a safety assessment for (nano)drug development. Many commercial oxidative stress assays have been developed for the evaluation of different stress pathways, such as ROS, lipid peroxidation, or consumption of antioxidants. A common cell‐based assay to measure non‐specific ROS generation in the nanotoxicology and nanomedicine field is H_2_DCF‐DA (2′,7′‐dichlorodihydrofluorescein‐diacetat).^[^ ^174^ ^]^ In the nanomedicine field it is crucial to demonstrate the inability of the NPs to induce higher ROS release in healthy off‐target tissues after the expected systemic exposure. Only few of the studies investigated oxidative stress responses (Table 2). Among the assessed nanozymes, only PEG‐Cu‐HCF nanozymes showed a slight increased ROS generation in the H_2_DCF‐DA assay but no GSH depletion or H_2_O_2_ production indicating the absence of an oxidative stress response under the investigated exposure conditions.^[^ ^169^ ^]^ Overall, the currently investigated nanozymes did not elicit off‐target oxidative damage in healthy cells.

### 3.4 Cell Death

Simply put, cell death, a fundamental biological process, halts of essential cellular functions resulting in cell demise. Regulated cell death, in addition to regulated cell proliferation, is necessary for the proper maintenance, function, and homeostasis of a living organism. Based on the evidence of the current nanomedicine research, cell death assessment is mostly addressed through live‐dead cell discrimination. Different NP properties seem to be able to influence the cell death mechanisms. One characteristic that can play a significant role is the NP dissolution and whether metal ions or the particulate form activate different cell death mechanisms. Rohde et al. discovered that silver (Ag) NPs prompted lipid peroxidation, whereas Ag^+^ ions elicited oxidative stress, leading to necrotic and apoptotic cell death, respectively.^[^ ^175^ ^]^ Furthermore, concentration and surface functionalization of NPs may also alter the cell death‐dependent fate.^[^ ^176^, ^177^ ^]^ Gallud and coworkers demonstrated that amine‐modified Au‐NPs at low concentration initiated the apoptotic pathway, whereas high concentration of amine‐modified Au‐NPs triggered the necroptotic pathway.^[^ ^176^ ^]^ In addition, the apoptosis‐necrosis transition triggered by Au nanorods was dependent on the density of the surface molecule, where small surface density molecule induced mostly apoptosis, while large density molecules prompted necrosis.^[^ ^177^ ^]^ Hence, nanozymes, as a subclass of nanomedicines should also be evaluated for their ability to activate different cell death mechanisms. Four nanozymes, namely Cu@MoS_2_‐PEG, PEG‐Cu‐HCF, PAA‐Fe_3_O_4_@GOx, and PVP‐coated Ir NPs with distinct therapeutic potential (tumor cell multiplication inhibition, tumor therapy, wound infection therapy, and acute kidney injury therapy, respectively) displayed no or negligible signs of apoptosis in healthy cells after 24 h of exposure.^[^ ^169^, ^178^, ^179^, ^180^ ^]^ In contrast, ultrasmall and biocompatibility layer‐lacking RuO_2_ induced an increase in the apoptotic cell population at a relatively low concentration (20 µg mL^−1^).^[^ ^181^ ^]^ Interestingly, the absence of a polymer coating (e.g., PEG, PAA) on the nanozymes appeared to show a positive correlation with apoptosis activation indicating that surface modifications strategies could be exploited to improve the safety profile of nanozymes. Different polymer‐, protein‐ and cell‐membrane based surface modifications have already been introduced and appraised for the improvement of biocompatibility, stability, and biodegradability of nanozymes without compromising their catalytic activity.^[^ ^182^ ^]^

### 3.5 Inflammation

Inflammation, under physiological conditions, is a pivotal mechanism for the protection and proper function of the human body. However, excessive inflammation can be detrimental and lead to pathogenesis. It is therefore necessary to examine whether nanozymes can have the potency to cause profound inflammatory responses.

Liu and colleagues engineered a Co‐doped Fe_3_O_4_ nanozyme with the capacity to scavenge both reactive oxygen and nitrogen species (RONS) in a post‐ischemic stroke reperfusion injury.^[^ ^183^ ^]^ The nanozyme was challenged against two in vivo stroke models (photothrombotic stroke and transient middle cerebral artery occlusion stroke) and successfully waned the infarct volume in both stroke models. In vitro, administration of the nanozyme in neuronal cells did not elicit any pro‐inflammatory cytokines (TNF‐α, IL‐6, and IL1‐β) suggesting an anti‐inflammatory behavior in healthy cerebral regions when counteracting stroke‐induced neuroinflammation.^[^ ^183^ ^]^ Likewise, Prussian blue (PB) nanozymes synthesized for the promotion of skin wound healing, did not elevate the levels of pro‐inflammatory mediators (TNF‐α and IL1‐β) in murine macrophages.^[^ ^184^ ^]^ While data from these two nanozymes with different therapeutic action areas is encouraging, more research is needed to confirm the non‐inflammatory profile for other types of nanozymes.

### 3.6 Mitochondrial Activity

Mitochondria are fundamental for the survival and the proper function of a cell, given their vital activities for energy production, metabolism, and cell signaling. As a result, malfunctioned mitochondria are the ground zero for a broad range of diseases, covering from cardiovascular to neurodegenerative.^[^ ^185^ ^]^ Mitochondrial dysfunction can originate from aberrant biogenesis, mitochondrial unfolded protein response, dynamic fission‐fusion, or mitophagy.^[^ ^186^ ^]^ There is a strong body of evidence on the interaction of NPs and mitochondria and their subsequent negative impact on the physiological mitochondrial processes. For instance, carbon black and silica NPs can impede mitochondrial biogenesis, fusion, and fission, leading to osteogenic differentiation inhibition and endothelial dysfunction, respectively.^[^ ^187^, ^188^ ^]^ Furthermore, NPs such as TiO_2_ and fullerene, can also interfere with the electron transfer chain by obstructing the mitochondrial membrane potential (MMP) and generating ROS.^[^ ^189^, ^190^ ^]^ Given the significance of mitochondria in the cell physiology and their relevance as a potential target for nanotoxicity, it is imperative to address the basic mitochondrial functionality markers (ATP production, MMP) during the safety assessment of nanomedicines. In the field of therapeutic nanozymes, data on the potential adverse effects on mitochondrial functions are scarce. Ruthenium oxide (RuO_2_) nanozymes, which have been investigated for their potential in preventing acute kidney injury, did not interfere with the MMP of HEK‐293 cells after 24 h of exposure.^[^ ^181^ ^]^ More research is warranted to understand nanozyme‐mitochondria interactions.

## 4 In Vivo Safety Landscape of Nanozymes

In vivo general toxicity testing covers an extensive endpoint battery, such as monitoring animal in‐life changes (clinical observations, body weight, food, water consumption, etc.), clinical pathology (hematology, blood chemistry, urine analysis, etc.), toxicokinetics (ADME parameters) and pathology (macroscopic, organ weights, microscopic, etc.). The above‐mentioned endpoints provide insights on the general health and welfare of the animal after exposure to the chemical in question.

**Figure 4:**
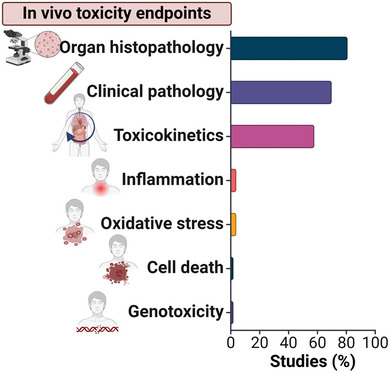
In vivo toxicological endpoints addressed in scientific studies evaluating the therapeutic potential of nanozymes.

The safety evaluation in the current in vivo studies mostly addressed the organism pathophysiology focusing on organ histopathology, clinical pathology, and toxicokinetics, as well as inflammation, oxidative stress, cell death, and genotoxicity (**Figure** **4**). All in vivo studies reported in this review were performed with mice as animal model, except from one study focusing on novel therapies to treat inflammatory bowel disease. This study, in addition to mice, used dogs, since they (as other larger mammals) are better representatives of the human gastrointestinal (GI) physiology and thus more suitable for translational research.^[^ ^218^ ^]^ In addition, nearly all studies, with very few exceptions, considered body weight as a non‐specific marker of general animal health and well‐being and demonstrated that it was not affected by the nanozyme administration.

### 4.1 Histopathology of Major Organs

Currently, in vivo studies investigating the therapeutic potential and the proof‐of‐concept of nanozymes, conduct a histopathology examination to prove the in vivo biocompatibility of the nanozymes. The heart, lungs, liver, kidneys, and spleen are considered vital organs for organism survival and are therefore at the forefront of toxicity evaluation. Most of the published literature investigated the nanozyme biocompatibility in these major organs, with few exceptions including additional organs such as brain, gastrointestinal tract (GIT), and pancreas (**Table** **3**). Collectively, the preclinical studies in mice did not show histological abnormalities from different nanozymes including different administration route [IV, subcutaneous (SC), intraperitoneal (IP), intratumoral (ITu), and oral]. However, the duration of exposure in all studies did not exceed the subacute classification (14‐28 days) acquired by regulatory authorities. Consequently, long‐term safety remains to be confirmed.

**Table 3:** Studies conducting in vivo toxicity assessment of therapeutic nanozymes. Normal font: no toxic effect, italic font: mild to moderate effect.

Material	Nanozyme	Size [nm]	Activity	Animal model/ strain/ sex/ age	Organ pathology/ dose/ admin. route/ time post exposure/ outcome	Clinical pathology components/ dose/ admin. route/ time post exposure/ outcome	ADME Parameters/ dose/ admin. route/ time post exposure/ outcome	Other endpoints/Assay/ dose/ admin. route/ time post exposure/ outcome	Ref.
Metals	Au_1_Pd_3_‐FA Cancer	2–3	SOD MPO	Mouse BALB/c Female 6–8 weeks	Heart, liver, spleen, lung, kidney, brain/ 0.3 mmol kg^−1^/ IV every 2 days for a total of 4 injections/ 7, 28 days/ no morphological abnormality	WBC, LYM, HB, MON, GRA, MCH, RBC, MCV, HCT, RDW‐SD, RDW‐CV, PLT, PCT, MPV, PDW, P‐LCR, ALT, AST, ALP, BUN, CRE/ 0.3 mmol kg^−1^/ IV every 2 days for a total of 4 injections/ 7, 28 days/ no significant alterations	Distribution, blood ½‐life, excretion/ 0.3 mmol/kg/ IV/ distribution: 2, 4, 12, 24, 48 h, blood ½‐life: 10 min – 36 h, excretion: 0.5, 1, 2, 4, 7 days/ distribution: kidney > liver > tumor > brain > lung > heart > spleen, blood ½‐life: ≈2.12 h, excretion: substantial clearance after 7 days, mostly eliminated through urine.	NA	[46]
	Ag@Pt‐NIL/HA Liver fibrosis	60–70	SOD CAT	Mouse ICR Male ND	Heart, liver, spleen, lungs, kidney/ 2, 5, 10 mg kg^−1^/ IV thrice a week/ 8 weeks/ no pathological features	AST, ALT, ALP, ALB, TP, BUN, CRE, UA/ 2, 5, 10 mg kg^−1^/ IV/ 1, 2, 4, 6, 8, 12, 24 h/ no significant change	Distribution, excretion/ 5 mg kg^−1^/ IV/ distribution: 12, 24 h, excretion: 6, 12, 24 h/ distribution: liver > spleen > lung > kidney > heart, excretion: mostly excreted by feces (max. amount at 12 h)	NA	[191]
	Cu_2‐x_Se‐PEG‐GOx Cancer	110.1–127.5	CAT GOx POD GSH OXD	Mouse BALB/c Female 6 weeks	heart, liver, spleen, lung, kidney/ 20 mg kg^−1^/ IV/ 14 days/ no evident injury	ALT, ALP, AST, BUN, CRE, WBC, RBC, HB, HCT, MCV, MCHC, MCH/ 20 mg kg^−1^/ IV/ 14 days/ no obvious distinctions	Distribution/ 20 mg kg^−1^/ IV/ 1, 8 h, 1, 2, 7 days/ liver > spleen > tumor > kidney > lung > heart	NA	[192]
	Pd@Pt‐T790 Bacterial infection	50	CAT	Mouse BALB/c Male ND	Heart, liver, spleen, lung, kidney/ ND/ IV/ 14 days/ no noticeable signs of damage	RBC, WBC, GRAN, PLT, LYM, MID, HB, ALT, AST, CK, BUN, CRE/ ND/ IV/ 14 days/ no obvious differences	NA	NA	[193]
	Zr^4+^‐Ru^3+^/Pt^4+^‐Ce6@HA Cancer	125	CAT POD GSH OXD	Mouse BALB/c Male 5 weeks	Heart, liver, spleen, lung, kidney/ 15 mg kg^−1^/ IV on day 0, 2, 4/ 15 days/ no obvious inflammation and organ abnormality	TP, BUN, GLB, ALT, ALB, A/G, AST, ALP, ALT/AST/ 15 mg kg^−1^/ IV on day 0, 2, 4/ 15 days/ normal function	Distribution, blood ½‐life/ 15 mg kg^−1^/ IV/ distribution: 0.5, 2, 6, 12, 24, 48 h, blood ½‐life: 5 min – 24 h/ distribution: ND, blood ½‐life: 2.6 h	NA	[194]
	Se@SiO_2_–Mn@Au/DOX Cancer	120	GOx POD	Mouse Kumming, Sprague Dawley ND ND	Heart, liver, spleen, lung, kidney/ 2 mg mL^−1^/ ND/ 14 days/ no significant difference	NA	Distribution, blood ½‐life, excretion/ 20 mg kg^−1^/ IV/ distribution: 2–48 h, blood ½‐life: 5 min – 24 h excretion: 12 h – 15 days/ distribution: liver > spleen > kidney > lung > heart > tumor, blood ½‐life: 2.18 h, excretion: mainly through urine (max between day 3 and 9)	NA	[195]
	Fe@melittin pro‐peptide Cancer	≈124	POD OXD GSH OXD	Mouse BALB/c Female 6 weeks	Heart, liver, spleen, lung, kidney/ 20 mg kg^−1^/ IV on day 0, 3, 5/ 9 days/ structurally intact	ALT, AST, CRE, WBC, HB, MCH, MCHC, RBC, MCV, HCT/ 20 mg kg^−1^/ IV on day 0, 3, 5/ 9 days/ normal indicators	NA	NA	[196]
Metal oxides	DEX‐CeO_2_ Urinary tract infection	2–3	SOD CAT RNS scavenger	Mouse C57BL/6J Female 6–8 weeks	Heart, liver, spleen, lung, kidney/ 8 mg kg^−1^/ IV 3 times a week/ immediately/ negligible toxicity	NA	Distribution, excretion/ 8 mg kg^−1^/ IV/ distribution: ND, excretion: 4 h/ distribution: spleen > liver > lung > kidney > heart, excretion: through urine	NA	[41]
	PAA‐Fe_3_O_4_@GOx Skin injury	289.2	GOx POD	Mouse BALB/c Female NA	Heart, liver, spleen, lung, kidney/ 200 µg mL^−1^/ in situ SC/ 8 days/ no obvious abnormalities	NA	NA	NA	[178]
	RuO_2_ Kidney injury	≈2	SOD CAT POD GPx	Mouse ICR Female 4–6 weeks	Heart, liver, spleen, lung, brain, intestine, kidney (tubules, collecting ducts, glomerulus, urethra)/ 5 mg kg^−1^/ IV/ 7 days/ no significant damage	BUN, CRE, CK, MDA/ 5 mg kg^−1^/ IV/ 1, 2, 3, 7 days/ no changes compared to control mice	Distribution, blood ½‐life, excretion/ 5 mg kg^−1^/ IV/ distribution: 2–72 h, 7 days, blood ½‐life: 2–24 h, excretion: 4, 8, 12 h, 1–7 days/ distribution: lung > liver > spleen > heart ≈ brain, blood ½‐life: 1.58 h, excretion: most of the RuO_2_ were excreted by urine in the first 12 h, followed by fecal excretion from day 1–7.	Oxidative stress: H_2_DCF‐DA/ 5 mg kg^−1^/ IV/ 24 h/ no oxidative damage in renal tissues	[181]
	Au‐DNA‐Fe_3_O_4_ Cancer	712	GOx POD	Mouse Kumming Female ND	Heart, liver, spleen, lung, kidney/ 10 mg kg^−1^/ IV/ 28 days/ no abnormalities	ALT, AST, ALB, GLB, TP, BUN, CRE, RBC, WBC, PLT, MCH, MCHC, MCV, HCT, HB/ 10 mg kg^−1^/ IV/ 0, 7, 28 days/ no obvious toxicity	Distribution/ 10 mg kg^−1^/ IV/ 4, 24, 48 h/ liver > spleen > lung > kidney > tumor > heart (all time points).	NA	[199]
	OA‐MnO_2_ Bacterial infection + Skin injury	50	POD OXD	Mouse BALB/c Female 6 weeks	Heart, liver, spleen, lung, kidney/ 8 mm/ ND/ once per day for 6 days/ non‐detectable toxicity	NA	NA	NA	[200]
	Fe_3_O_4_/Ag/Bi_2_MoO_6_ Cancer	35	SOD CAT POD GSH OXD	Mouse BALB/c Female 5–6 weeks	Heart, liver, spleen, lung, kidney/ 100 µg mL^−1^/ IV/ NA/ no noticeable damage	ALT, AST, ALP, BUN, CRE, WBC, RBC, HB, HCT, MCV, MCH, MCHC, RDW, PLT, MPV, PDW/ 100 µg mL^−1^/ IV/ ND/ no abnormality	Distribution, blood ½‐life/ 100 µg mL^−1^/ IV/ distribution: 6, 8, 10, 12, 24 h, blood ½‐life: 1–24 h/ distribution: liver > spleen > lung > tumor > kidney > heart, blood ½‐life: 3.15 h	NA	[201]
	SNLP‐IrO_x_‐BS12 Bacterial infection	37	POD	Mouse ICR ND ND	Heart, liver, spleen, lungs, kidney/ 5 mg kg^−1^/ IV/ 24 h/ no significant changes	NA	NA	NA	[203]
	Fe_3_O_4_‑GOx Diabetic ulcer	13	CAT POD GOx	Mouse Type II diabetic ND 6 weeks	Heart, liver, spleen, lung, kidney/ 200 µg mL^−1^/ SC for 7 days/ immediately/ no lesions or inflammation	TP, ALP, ALB, ALT, GLB, AST, A/G, BUN/ 200 µg mL^−1^/ SC for 7 days/ immediately/ no obvious difference	NA	NA	[204]
	CeO_2_‐PA Cancer	ND	SOD CAT	Mouse BALB/c nude Male 8 weeks	Heart, liver, spleen, lung, kidney/ 5 mg kg^−1^/ IV at 1st and 8th day/ 14 days of therapy/ no obvious abnormalities	WBC, RBC, HB/ 5 mg kg^−1^/ IV on day 1 and 8/ 14 days of therapy / no obvious abnormalities	Distribution/ 5 mg kg^−1^/ IV/ 1, 7 days/ liver > spleen > kidney ≈ tumor > heart > lung	NA	[205]
	Cu_x_O@EM‐K Alzheimer's	≈70	SOD CAT GPx	Mouse C57BL/6 ND ND	Heart, liver, spleen, lung, kidney/ 15 mg kg^−1^/ IV for 20 days/ immediately/ no obvious signal of organ harm	ALT, AST, ALP, BUN, CRE, RBC, WBC, PLT, TP, ALB, HB, MCH/ 15 mg kg^−1^/ IV for 20 days/ immediately/ no noticeable changes	Distribution, blood ½‐life/ 15 mg kg^−1^/ IV/ ND/ distribution: liver > spleen > lung > kidney > heart > brain, blood ½‐life: ND	NA	[206]
	IR780‐MnO_2_‐PLGA Cancer	166	HPOD GSH OXD	Mouse BALB/c nude Female 6 weeks	Heart, liver, spleen, lung, kidney, brain/ 2 mg mL^−1^/ IV/ 1, 7, 14, 28 days/ no apparent damage	ALT, AST, BUN, CK, WBC, MCV, HB/ 2 mg mL^−1^/ IV/ 1, 7, 14, 28 days/ negligible differences	Distribution/ 2 mg mL^−1^/ IV/ 4 h/ tumor > liver > spleen > lung > kidney > heart	NA	[207]
	Citrate‐Mn_3_O_4_ Huntington's	≈6	POD GPx	Mouse C57BL/6J ND 6–8 weeks	NA	HB, RBC, RT, HCT, MCV, MCH, MCHC, PLT, WBC, LYM, PT, NEU, MON, EOS, BAS APTT/ 0.5 mg kg^−1^/ IP for 16 days/ immediately/ insignificant changes	NA	NA	[208]
	Cu_2_MoS_4_ Bacterial infection	≈28	POD OXD	Mouse BALB/c Female ND	Heart, liver, spleen, lung, kidney/ 8 mg kg^−1^/ IV/ 14 days/ no significant abnormality or damage	NA	NA	NA	[209]
	CeO Acute inflammation	2.8	SOD CAT	Mouse C57BL/6J Male 12 weeks	Heart, liver, spleen, lung, kidney/ 100 mg kg^−1^/ IV/ 24 h/ no noticeable signs of acute toxicity	ALT, AST, TB, BA, BUN/ 100 mg kg^−1^/ IV/ 24 h/ no statically significant differences	Distribution, blood ½‐life/ 100 mg kg^−1^/ IV/ 24 h/ distribution: liver > spleen > kidney > lung > heart, blood ½‐life: 29 min	NA	[210]
	MnOx‐coated Au Cancer	≈150	NADPH OXD POD	Mouse BALB/c Female ND	Heart, liver, spleen, lung, kidney/ ND/ IV on day 0 and 7/ 7 days/ no noticeable damage	ALT, AST, AST/ALT, TP, ALB, GLB, A/G, GT, BUN, UA, CRE/ ND/ IV on day 0 and 7/ 7 days/ no significant damage	Distribution, blood ½‐life/ NA/ IV/ distribution: 6, 12, 24, 48 h, blood ½‐life: 30 min – 24 h/ liver > spleen ≈ lung > tumor > heart > kidney, blood ½‐life: 1.325 h	NA	[211]
Carbons	CDs Ischemic stroke	2	SOD	Mouse C57BL/6J Male 8–10 weeks	Heart, liver, spleen, lung, kidney, brain/ 2.5 mg kg^−1^/ IV/ 7 and 30 days/ no signs of damage	ALP, AST, CRE, BUN, ALT, WBC, LYM, MON, GRA, HGB, MCH, MCHC, RBC, MCV, HCT, RDW‐SD, RDW‐CV, PLT, PCT, MPV, PDW, P‐LCR/ 2.5 mg kg^−1^/ IV/ 7 and 30 days/ no significant difference	Distribution, blood ½‐life/ 2.5 mg kg^−1^/ IV/ distribution: 2, 6, 24 h, blood ½‐life:5 min – 12 h/ distribution: liver > kidney > lung ≈ spleen ≈ heart ≈ brain, blood ½‐life: 53 min	NA	[50]
	CDs Lung injury	2.7±0.7	SOD RONS scavenger	Mouse C57BL/6J Male 6–8 weeks	Heart, liver, spleen, lung, kidney/ 20 mg kg^−1^/ IP/ 7 and 30 days/ no visible damage	ALT, AST, ALP, BUN, CRE, wide range of blood test parameters/ 20 mg kg^−1^/ IP/ 7 and 30 days/ all in normal levels	Distribution/ 5 mg kg^−1^/ IP/ 1, 2, 4, 8, 24 h/ 1, 2, 4, 8 h: kidney > liver > lung > heart > spleen, 24 h: liver > kidney > lung > heart > spleen	NA	[213]
	CDs Inflammatory bowel disease	3	SOD ^●^OH scavenger	Mouse C57BL/6 ND ND	Heart, liver, spleen, lung, kidney/ 1, 5, 10 mg kg^−1^/ IV every other day three times/ 8 and 30 days/ no structure and pathological changes	WBC, RBC, PLT, HB, GRAN, LYM, AST, ALT, BUN, CRE, CK/ 1, 5, 10 mg kg^−1^/ IV every other day three times/ 8 and 30 days/ no apparent blood toxicity	Distribution, blood ½‐life/ 5 mg kg^−1^/ IV/ 5 min – 12 h/ distribution: kidney > liver > lung > colon > heart > spleen, blood ½‐life: 11.5 min	NA	[214]
	V_2_N MXene Bacterial infection	200	POD OXD	Mouse BALB/c Female 6 weeks	Heart, liver, spleen, lung, kidney/ 50 µg mL^−1^/ IP/ 10 days/ no obvious damages	ALT, AST, BUN, GT, CRE, MON, LYM, GRAN, RBC, WBC, MCV, PLT/ 50 µg mL^−1^/ IP/ 10 days/ no obvious abnormalities	NA	NA	[215]
**SAzymes**	**MOF‐based**								
	Ca/Fe Prussian Blue Pancreatitis	≈7.5	SOD POD GPx RONS scavenger	Mouse ICR Female 6–8 weeks	Heart, liver, lung, kidneys, pancreas/ 200 µg mL^−1^/ IV/ 30 days/ no significant morphological changes	AST, ALT, BUN, CRE, AMYL, LIP/ 200 µg mL^−1^/ IV/ 30 days/ normal range	NA	Inflammation: ELISA: TNF‐a, IL‐1β, IL‐6/ 200 µg mL^−1^/ IV/ 30 days/ normal range	[216]
	PEG‐MOF/PtAu Cancer	ND	CAT OXD	Mouse BALB/c Female 6 weeks	Heart, liver, spleen, lung, kidney/ 20 mg kg^−1^/ IV on day 2, 4, 7, 10/ 4 days post last injection/ no significant morphological difference	ALT, AST, BUN, CRE/ 20 mg kg^−1^/ IV on day 2, 4, 7, 10/ 4 days post last injection/ within the normal ranges	Distribution, blood ½‐life/ 20 mg kg^−1^/ IV/ distribution: 24 h, blood ½‐life: 30 min – 24 h/ distribution: tumor > liver > kidney > lung > spleen >heart, blood ½‐life: 1.71 h	NA	[217]
	DOX‐Fe@ZIF‐8 Cancer	230	POD	Mouse BALB/c Female ND	Heart, liver, spleen, lung, kidney/ 4 mg kg^−1^/ IV/ 7 days/ no apparent toxic effects for spleen lung and kidney. Heart and liver: the symptoms caused by the NPs were alleviated compared to pure DOX.	NA	Distribution/ 4 mg kg^−1^/ IV/ 12, 24 h/ nanozyme signal observed in the tumor site and abdominal region	NA	[224]
	Mn‐CDs@ZIF‐8@Au Cancer	135	CAT GOx	Mouse BALB/c Female 4–6 weeks	Heart, liver, spleen, lung, kidney/ 4 mg mL^−1^/ IV/ 24 h/ lack of identifiable histopathological damage	ALP, ALT, AST, BUN, GLU, WBC, RBC, HCT, MCV, MCHC, MCH, PLT, HB/ 4 mg mL^−1^/ IV/ 24 h/ absence of abnormalities	Distribution/ 4 mg mL^−1^/ IV/ 1, 3 days/ liver > spleen > tumor > kidney > lung > heart	NA	[231]
**Other**								
	PEG‐Cu‐HCF Cancer	102.5± 21.8	POD GSH OXD	Mouse BALB/c NA NA	Heart, liver, spleen, lung, kidney, intestine/ 5 mg kg^−1^/ IV+ITu/ 21 days/ no noticeable differences	*WBC, RBC, HB, HCT, MCV, MCH, MCHC, PLT, MPV, ALP, ALT, AST, BUN/ 5 mg kg^−1^/ IV+ITu/ 2 and 14 days/ after 2 days: ALP (2.1‐2.3 fold), AST (2.5‐2.7 fold) increase, 14 days: within the normal ranges*	NA	NA	[169]
	PVP‐Ir Kidney injury	1‐2	SOD CAT POD RNS scavenger	Mouse BALB/c Female ND	Heart, liver, spleen, lung, kidney (tubules, collecting duct, glomerulus, urethra)/ 50 mg kg^−1^/ IV/ 30 days/ no noticeable damage	ALT, AST, BUN, CRE/ 50 mg kg^−1^/ IV/ 30 days/ in the normal range	Distribution, blood ½‐life, excretion/ 5 mg kg^−1^/ IV/ 2, 4, 8, 12, 24 h/ distribution: kidney ≈ liver > spleen > lung > heart, blood ½‐life: 2.27 h, excretion: healthy > AKI mouse	**Inflammation**: ELISA: TNF‐a, IL‐6/ 50 mg kg^−1^/ IV/ 30 days/ no particular difference. **Oxidative stress**: TBARS: lipid peroxidation/ 3.3 mg mL^−1^/ IV/ 24 h/ no change, ELISA: HO‐1/ 3.3 mg mL^−1^/ IV/ 24 h/ no change. DHE/ NA/ IV/ ND/ no ∙O_2_^−^ generation, in renal tissues. **Genotoxicity**: ELISA: 8‐OHdG/ 3.3 mg mL^−1^/ IV/ 24 h/ no residues in renal tissues	[179]
	Cu@MoS_2_‐PEG Cancer	ND	POD	Mouse BALB/c ND ND	Heart, liver, spleen, lung, kidney/ 10 mg kg^−1^/ IV/ 15 days/ no evident alterations	WBC, LYM, MON, GRAN, RBC, HB, HCT, MCV, MCH, MCHC, RDW, PLT/ 10 mg kg^−1^/ IV/ 15 days/ within the reference range	NA	NA	[180]
	Co‐Fe_3_O_4_ Ischemic Stroke	45	SOD CAT RNS scavenger	Mouse C57BL/6J Male 8–10 weeks	Heart, liver, spleen, lung, kidney/ 2 mg kg^−1^/ IV/ 24 h and 72 h/ no signs of toxicity	NA	Distribution/ 2 mg kg^−1^/ IV/ 15 min – 72 h, 2 and 4 weeks/ 15 min – 1 h: kidney > liver, 2–4 h: kidney, 24 h: kidney >> brain, 72 h: kidney > liver > brain, 2 and 4 weeks: liver > brain. No nanozyme intensity was observed in heart, spleen, lung at any time point.	NA	[183]
	B‐SA_50_ Inflammatory bowel disease	168	SOD CAT ^●^OH scavenger	**Mouse** C57BL/6 Female 6–8 weeks **Dog** Beagle Male 40–45 weeks	**Mouse**: Heart, liver, spleen, lung, kidney, intestines, stomach/ B‐SA_50_: 500 µg mL^−1^/ oral administration for 28 days/ immediately/ no distinct difference **Dog**: Heart, liver, spleen, lung, kidney, esophagus, small intestine, stomach, jejunum, ileum/ B‐SA_50_: 0.5 mg kg^−1^/ oral administration for 6 days/ immediately/ negligible differences	**Mouse**: WBC, NEU, LYM, MON, BAS, RBC, HB, MCV, MCH, MCHC, RDW‐SD, PLT, MPV, PDW, PCT, HCT, ALT, AST, ALT/AST, TP, ALB, GLB, A/G, ALP, GGT, BUN, CRE, UA, GT, TBIL, DBIL, IBIL/ B‐SA_50_: 500 µg mL^−1^/ oral administration for 28 days/ immediately/ no distinct difference	NA	NA	[218]
	DSPE‐PEG_2000_‐RosA‐Mn Kidney injury	142	SOD CAT RONS scavenger	Mouse C57BL/6 Male 8 weeks	Heart, liver, spleen, lung, kidney/ 2 mg kg^−1^/ IV once per day for 3 days/ immediately/ no obvious congestion and necrosis	ALT, AST, CRE, BUN/ 2 mg kg^−1^/ IV once per day for 3 days/ immediately/ within the normal ranges	Distribution/ 1 mg mL^−1^/ IV/ 1, 3, 5, 10 h/ liver > kidney > lung > heart ≈ spleen (all time points).	Cell death: TUNEL/ 2 mg kg^−1^/ IV once per day for 3 days/ immediately/ no obvious apoptosis in kidney regions	[220]
	Pt‐PCN224‐GOx‐EM Cancer	156.9± 0.9	CAT GOx	Mouse BALB/c nude Female 3–4 weeks	Heart, liver, spleen, lung, kidney/ 10 mg/ kg/ IV/ 15 days/ no significant differences	ALT, AST, CRE, BUN/ 10 mg kg^−1^/ IV/ 15 days/ no obvious function damage or pathological alteration	Distribution, blood ½‐life/ 10 mg kg^−1^/ IV/ 3 min – 24 h/ distribution: liver > spleen > tumor > lung > kidney > heart, blood ½‐life: 4.21±0.17 h	NA	[221]
	PtCu_3_‐PEG Cancer	14	HRPOD GPx	Mouse BALB/c Female ND	Heart, liver, spleen, lung, kidney, stomach, intestine/ 10 mg kg^−1^/ IV for 1, 7, or 30 days/ ND/ no morphological change	RBC, WBC, HB, HCT, MCH, MCHC, MCV, PLT, MPV, AST, ALP, ALT, GT, CRE, BUN/ 10 mg kg^−1^/ IV for 1, 7 or 30 days/ ND/ no significant changes	Distribution/ 10 mg kg^−1^/ IV/ 24 h/ spleen > liver > tumor > lung > kidney > heart	NA	[222]
	BSA‐Cu Cancer	7.6±0.9	POD GSH OXD	Mouse BALB/c Male 4 weeks	Heart, liver, spleen, lung, kidney/ 2, 4 mg kg^−1^/ IV twice in 48 h intervals for 30 days/ ND/ no pathological changes	ALT, AST, BUN, BUN, LDH, WBC, RBC, LYM, GRAN, PLT, HCT/ 2, 4 mg kg^−1^/ IV twice in 48 h intervals for 30 days/ ND/ in the normal ranges	Distribution, blood ½‐life, excretion/ IV/ 0, 0.5, 2, 8, 12, 24, 48 h/ NPs were mostly observed in kidney and liver followed by the tumor, reaching a peak at 8 h, blood ½‐life: 1.55 h, excretion: renal clearance	NA	[223]
	BSA@CeO/Fe^2+^ Cancer	3.46	SOD CAT POD	Mouse BALB/c Female 4–6 weeks	Heart, liver, spleen, lung, kidney/ 4 mg kg^−1^/ IV on day 1, 7, 30/ immediately/ no visible organ damage or inflammation	*ALT, AST, BUN, A/G, WBC, RBC, HB, MCH, MCHC, PLT, HCT/ 4 mg kg^−1^/ IV on day 1, 7, 30/ no long‐term damage but short ‐term increase in PLT and WBC on day 1*	Distribution/ 4 mg kg^−1^/ IV/ 24 h/ Fe: spleen > heart > kidney > lung > liver, Ce: spleen > liver > kidney > lung > heart	NA	[225]
	SA‐Ce‐N_4_‐C‐OH_2_ Hyperglycemia	ND	SOD CAT POD OXD	Mouse C57BL/6J Male 6 weeks	Heart, liver, spleen, lung, kidney, SC fat, pancreas, testis, epididymis, ileum, colon viscera/ 10 mg kg^−1^/ IP every day for 4 weeks/ 12 h/ no obvious pathological damage	AST, ALT, BUN, CRE, TG, CHO, LDL, HDL, LDH, GLU/ 10 mg kg^−1^/ IP every day for 4 weeks/ 12 h/ no damage to liver biochemistry and function or kidney function	Distribution/ 10 mg kg^−1^/ IP every day for 4 weeks/ immediately/ muscle > liver > kidney > lung > brain > heart > epididymis ≈ blood/ IP/ 24, 48, 72 h/ liver > colon > ileum > kidney > brain > testis > lung ≈ muscle ≈ pancreas > spleen ≈ fat on inguinal, shoulder blade, visceral epididymis > heart	NA	[226]
	Cu‐DCA Diabetic skin injury	20–25	SOD CAT	Mouse BALB/c Male 8 weeks	Heart, liver, spleen, lung, kidney/ 24 µm/ topical on the skin wound on day 3, 7, 11, 14/ no damage or pathological changes	AST, ALT, BUN, CRE, BUN, RBC, WBC, HB, PLT, MCH/ 24 µm/ topical on the skin wound on day 3, 7, 11, 14/ within the normal range	NA	NA	[227]
Smart assembly‐Nano ‐hybrid	Arg‐Gly‐Asp‐GOx‐CAT Cancer	65	CAT GOx	Mouse BALB/c ND ND	Heart, liver, spleen, lung, kidney/ ND/ IV once every 2 days/ after the 14th day/ no distinct differences	NA	Distribution/ 1 mg mL^−1^/ IV/ 48 h/ tumor > liver > kidney > lung > heart ∼ spleen	NA	[229]

Abbreviations: Hematology: WBC: White blood cells, RBC: Red blood cells, LYM: Lymphocytes, GRAN: Granulocytes, MON: Monocytes, NEU: Neutrophils, EOS: Eosinophils, BAS: Basophils, RT: Reticulocytes, PLT: Platelets, MID: Intermediate cells, RDW: Red cell distribution width, RDW‐CV: Red cell distribution width – Coefficient of Variation, RDW‐SD: Red cell distribution width – Standard Deviation, MPV: Mean platelet volume, PDW: Platelet distribution width, P‐LCR: Platelet‐large cell ratio, HCT: Hematocrit, MCV: Mean corpuscular volume, HB: Hemoglobin, MCH: Mean corpuscular hemoglobin, MCHC: Mean corpuscular hemoglobin concentration, PT: Prothrombin time, APTT: Activated Partial Thromboplastin Time. Clinical chemistry: ALP: Alkaline phosphatase, ALT: Alanine transaminase, AST: Aspartate aminotransferase, BUN: Blood urea nitrogen, CRE: Creatinine, GT: γ‐glutamyl transferase, PCT: Procalcitonin, AMYL: Amylase, LIP: Lipase, ALB: Albumin, TP: Total protein, UA: Uric acid, GLB: Globulin, CK: Creatinine kinase, LDH: Lactate dehydrogenase, A/G: Albumin/Globulin ratio, TB: Total bilirubin, BA: bile acids, TG: Triglycerides, CHO: Cholesterol, LDL: Low‐density lipoprotein, TBIL: Total Bilirubin, DBIL: Direct Bilirubin, IBIL: Indirect Bilirubin, HDL: High‐density lipoprotein, GLU: Glucose, MDA: Malondialdehyde.

### 4.2 Clinical Pathology

Clinical pathology analysis in preclinical studies is critical as it includes measurements of biomarkers in body fluids, which can be correlated to disease, pathology, or toxicological events. Most of the studies on therapeutic nanozymes investigated either hematology [e.g., white blood cells (WBC), platelets (PLT)], clinical chemistry parameters [e.g., alanine aminotransferase (ALT), creatinine (CRE)] or a combination of those (Table 2). Overall, no significant alteration of such parameters was observed, suggesting a generally good in vivo biocompatibility of nanozymes. A study of Yang et al. on a multifunctional nanozyme to enhance breast cancer therapy through radiosensitization observed a transient increase in WBC and PLT at day 1 but values recovered to physiological levels at day 7.^[^ ^225^ ^]^ Similarly, a Cu‐based nanozyme for tumor therapy, caused a short‐term (day 2) increase in the hepatic enzymes alkaline phosphatase (ALP) and aspartate aminotransferase (AST), as their levels returned to the physiological range after day 14.^[^ ^169^ ^]^

### 4.3 ADME Parameters – Pharmacokinetics

Besides target organ toxicity or blood biomarker fluctuations, it is also important to address the biodistribution and clearance of the nanozymes in the organism and associate it with toxicological findings. Data from nanozyme‐mediated cancer treatment studies (Table 3) revealed a trend for the accumulation of a substantial amount of nanozymes in other organs besides the tumor tissue. As already known for other NP types, these included the reticuloendothelial system (RES – liver, spleen) and clearance organs (kidneys). Interestingly, nanozyme accumulation in the lungs, an organ also rich in phagocytic cells, was consistently observed. Lung concentrations were sometimes even higher than those in the spleen or kidney, indicating that future hazard assessment of therapeutic nanozymes should consider potential pulmonary toxicity.

### 4.4 Oxidative Stress

The induction of oxidative stress in vivo was investigated in two studies, both addressing the treatment of acute kidney injury. Zhang and colleagues, after single IV injection of ultrasmall PVP‐coated iridium nanozymes (3.3 mg mL^−1^) in mice, evaluated the levels of lipid peroxidation, heme oxygenase 1 (HO‐1), and superoxide anions (O_2_^−^) in renal tissues, without observing any alteration.^[^ ^179^ ^]^ Equally, Liu et al. after single IV injection of ultrasmall RuO_2_ nanozymes (5 mg kg^−1^) confirmed the absence of oxidative damage (H_2_DCF‐DA assay) in renal tissues.^[^ ^181^ ^]^

### 4.5 Cell Death

Among the many types of cell death (apoptosis, necrosis, pyroptosis, ferroptosis), only apoptosis was addressed in vivo. Yuan et al. used a phenolic ligand, namely rosmarinic acid (RosA), to fabricate RosA‐Mn (DSPE‐PEG_2000_‐RosA‐Mn) nanozymes with antioxidant properties in order to mitigate acute kidney injury and further improve kidney functions.^[^ ^220^ ^]^ DSPE‐PEG_2000_‐RosA‐Mn nanozymes were IV administered (2 mg kg^−1^) for three days to healthy mice and revealed no evidence of apoptosis‐inducing potential in renal regions.

### 4.6 Inflammation

Inflammation in animals was determined by measuring the levels of inflammatory markers in the blood. Li et al. engineered Ca/Fe‐based PB nanozymes with an antioxidant profile in order to resolve inflammation in the pancreas and revealed no alterations in the physiological levels of pro‐inflammatory markers (TNF‐a, IL‐1β, IL‐6) residing in the blood.^[^ ^216^ ^]^ Likewise, no pro‐inflammatory marker (TNF‐a, IL‐6) activation was observed after PVP‐coated iridium nanozyme administration in the blood for kidney injury treatment.^[^ ^179^ ^]^ In both studies, the animals underwent a 30‐day nanodrug dosing regimen.

### 4.7 Genotoxicity

Genotoxicity refers to a genetic material damage caused by a noxious substance and includes gene mutations, DNA damage, or chromosomal aberrations. Primary direct genotoxicity encompasses the translocation of NPs into the nucleus and the subsequent impairment of DNA or chromosomes. Conversely, primary indirect genotoxicity is mediated through NP interactions with cell cycle components (i.e., mitotic spindle, DNA‐related proteins, responsible for replication, repair, etc. or proteins responsible for cell cycle regulation and its checkpoints) or through NP‐induced excessive ROS, resulting in DNA strand breaks or base lesions.^[^ ^230^ ^]^ Secondary genotoxicity is caused due to a NP‐induced inflammatory response, where the release of ROS and cytokines from immune cells can cause genotoxicity to neighbouring cells.^[^ ^230^ ^]^ To evaluate genotoxicity, there is a comprehensive battery of genotoxic assays, which covers a wide range of genotoxic endpoints, ranging from DNA breaks and mutations to micronucleus formation. From the pool of reviewed studies, only one investigated the genotoxic potential of therapeutic nanozymes. Specifically, PVP‐coated iridium nanozymes, engineered for acute kidney injury management, did not induce the formation of 8‐hydroxydeoxy guanosine (8‐OHdG – a biomarker of ROS‐driven DNA base modification) residues after a single IV dose (analysis at day 30 post‐treatment) to female mice.^[^ ^179^ ^]^

## 5 Conclusions on the Current Safety Profile of Therapeutic Nanozymes

Nanozymes have recently emerged as auspicious nanotherapeutics with versatile enzyme‐mimetic activities that can be tuned based on their morphology, chemistry, and smart design. Among these, SAzymes stand out as their structures have been found to closely mimic the catalytically active sites of natural enzymes. A variety of nanozymes have been reported since their discovery and the field has shifted from in vitro to in vivo studies, which signifies the potential of nanozymes in the treatment of various diseases involving oxidative stress and pathological inflammatory conditions. At this early stage of biomedical nanozyme development, exploitation of the therapeutic potential and identification of application domains for various diseases is often the main focus of preclinical studies. However, comprehensive hazard profiling is equally important to inform the safe design of the nanozymes and to prevent failure at a later stage of clinical transfer.

In general, the current research suggests the biocompatible character of therapeutic nanozymes regardless of their enzymatic activities but further data are needed for a thorough hazard assessment. Although we only covered an exemplary fraction of the identified literature reporting toxicity data of nanozymes, we found that the selected works performed a highly similar safety investigations approach. The most frequently studied in vitro endpoints were cell viability, hemolysis, and oxidative stress responses, whereas in vivo endpoints included organ pathology, clinical pathology, and ADME parameters. Inflammatory responses were rarely covered in vitro and in vivo. However, these parameters represent only a fraction of possible adverse bioresponses previously described for other NPs and may not capture more sublethal effects resulting in delayed disease development. For instance, immunomodulatory responses, genotoxicity, endocrine, and bio‐barrier disruption, or potential interference of nanozymes with gene expression, protein function, metabolism, or the microbiome are warranted to exclude delayed disease development. Further, long‐term toxicity evaluation is often overlooked, but its contribution is crucial for a holistic toxicity assessment to detect late‐onset adverse effects.^[^ ^232^, ^233^ ^]^ For example, prolonged NP risk assessments have demonstrated the NP potential to cause long‐term reproductive and pulmonary toxicity.^[^ ^234^, ^235^, ^236^ ^]^ Moreover, for metal‐containing nanozymes it will be crucial to provide more detailed insights on their biodegradation, potential release of metal ions, and subsequent metal‐induced toxicities. In addition, the literature overview has shown that several nanozymes have the potential for adverse hemolytic activity that should be carefully addressed. However, many studies did not follow the hemolytic criteria defined by the ASTM E2524‐08 standard and/or did not properly report important experimental parameters (e.g., incubation period), which impedes data interpretation and comparison. Absence of documentation of important experimental parameters (e.g., dose, exposure time, and administration route) was also observed for other endpoints, both in vitro and in vivo. This exemplifies the general need for standardization and harmonization of hazard assessment methods to achieve a better comparability, reliability, and robustness of results across studies. Notably, studies exploiting the promising potential of SAzymes, did not show any higher toxicity compared to conventional nanozymes, thus advertising their superior catalytic properties without compromising their biosafety. SAzymes are generally more stable and safer than all other kinds of nanozymes as the metal atom is in a stable coordination environment similar to a natural enzyme, not allowing the metal to leach in biological medium, thus posing less toxicity issues.^[^ ^65^, ^237^ ^]^

## 6 Future Research Directions

### 6.1 Emerging Nanomedicines Regulatory Requirements

**Figure 5:**
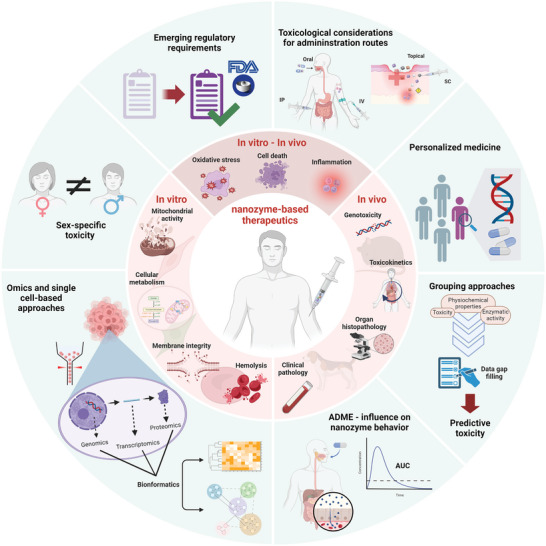
Current pre‐clinical safety assessment of therapeutic nanozymes and future research directions.

Nanozymes, as a potential new subclass of nanomedicines, must undergo regulatory evaluation according to regulatory standards to proceed toward clinical translation (**Figure** **5**). Nevertheless, no regulatory framework for nanotechnologies intended as therapeutics has been developed yet, rendering the existing regulatory guidance for common medicinal products inadequate. The European Union, trying to address this urgent regulatory issue, has recently (2019) published a document summarizing the main regulatory challenges and needs in the field of nanotechnologies intended as therapeutics.^[^ ^238^ ^]^ While there is no exhaustive set of regulations, this document may act as a compass to address some quality and safety aspects. Further, in 2022 the FDA delivered a nanomedicine guidance draft, which discusses considerations regarding quality of nonclinical and clinical studies.^[^ ^239^ ^]^ Despite the lack of regulatory requirements, almost 500 nanodrug clinical trials were identified during 2002–2021, indicating the urgent need of a regulatory review process.^[^ ^240^ ^]^ Interestingly, PB which has shown great potential as a multinanozyme mimetic^[^ ^241^ ^]^ toward the treatment of various pathological conditions (i.e., skin wounds and pancreatitis),^[^ ^184^, ^216^ ^]^ has already been approved by FDA as therapeutic agent (brand name: Radiogardase) for the treatment of internal contamination of cesium (radioactive) and thallium [(non‐)radioactive].^[^ ^242^ ^]^ Equally, another FDA‐approved drug,^[^ ^243^ ^]^ namely Ferumoxytol (iron oxide NP formulation, Brand name: Feraheme), used for the treatment of iron deficiency anemia, has recently shown that it can exert POD‐like activity for the eradication of cariogenic biofilms in human mouths.^[^ ^244^ ^]^ Even though the already FDA‐approved therapeutics have different purpose, mechanism of action, and indication compared to the nanozymes, they could in the future facilitate the development of a new iron oxide NP‐ or PB‐based nanodrug indication.

The Nanotechnology Characterization Laboratory of the National Cancer Institute (US‐NCL) as well as the European Nanomedicine Characterization Laboratory (EU‐NCL) have the capacity to provide scientific data for regulatory purposes of nanomedicines regarding their correlation between physicochemical properties and biological effects.

The NCLs provide a comprehensive assay cascade, which characterizes the nanotechnology's physicochemical properties and in vitro/in vivo compatibility. In vitro assays cover immunology, pharmacology, and toxicology, targeting a wide range of endpoints (e.g., platelet aggregation, oxidative stress, and phagocytosis). Similarly, in vivo studies include immunotoxicity, toxicokinetics, target organ acute and repeat‐dose toxicity as well as efficacy of the nanotechnology in question.

### 6.2 Understanding the ADME Parameters

Evaluation of ADME parameters is an indispensable component of the toxicological evaluation of a new (nano)drug (Figure 5). Augmented bioavailability, optimized penetration in target organs/tissues, and prolonged circulation times are some of the nanodrug characteristics that have attracted attention, given the dramatic differences compared to conventional drugs.^[^ ^245^ ^]^ A striking difference between nanomedicines and classical drugs is the circulation time, which is dependent on the immune recognition and the removal of NPs from the blood, compared to the concentration gradient of the drug molecule. Nanoformulations have been successfully used to improve ADME parameters of conventional drugs, aiming for lower dosage, administration frequency and thus improved toxicity profiles.^[^ ^245^ ^]^ Yet, adaptations in the nanozyme design to achieve prolonged blood circulation times and targeted biodistribution may turn out to be a double‐edged sword leading to an extended nanomedicine load in plasma or tissues, threatening the organism with nano‐related safety concerns. It is encouraging that the current toxicokinetics and safety studies in nanozyme research use the same administration route and dose as the efficacy studies, allowing the alignment of toxicity evaluation with the therapeutic efficacy and thus the investigation of a possible trend in the nanozyme behavior in the whole organism. This situation could allow a guided steering of nanozyme synthesis, toward the necessary ADME parameters for a specific therapeutic goal. US‐NCL has established general guidelines and recommendations for the execution of pharmacokinetic studies for two different animal models (mouse, rat) rendering it also more convenient for research groups that have access to only one of them. Consistent usage or consideration of these guidelines could pave the way toward the correlation of NP physicochemical characteristics and fate in the body. Nevertheless, it is worth to mention that, while US‐NCL in vivo guidelines and recommendations are not therapy‐wide applicable, but rather tumor therapy‐specific, they could still provide some standardization foundations across studies.

### 6.3 Pharmacokinetic Influence on Nanozyme Behavior

Pharmacokinetics can have a significant impact on the safety and therapeutic efficacy of nanozymes (Figure 5). As soon as nanomedicines are exposed to a biological environment (blood, microbes, cells, or biobarriers), they can be subject to biotransformation processes, including agglomeration/aggregation, decomposition, and protein corona formation.^[^ ^246^ ^]^

Agglomeration/aggregation can help mitigate the toxicity by reducing the cellular uptake and decreasing the active surface‐to‐volume ratio of NPs, which in turn alters ROS generation.^[^ ^247^, ^248^, ^249^ ^]^ Furthermore, the decomposition of NPs is another critical factor influencing their toxicity potential, primarily through the dissolution of ions from metallic NPs, such as metal oxides.^[^ ^175^, ^250^, ^251^ ^]^ For instance, after endocytosis, NPs may translocate to acidic subcellular compartments like lysosomes, where they undergo extensive degradation, resulting in the release of toxic ions – a process known as the Trojan horse mechanism.^[^ ^252^ ^]^ In addition, due to the high surface‐to‐volume ratio, NPs are prone to interact and adsorb a multitude of biomolecules (e.g., proteins, carbohydrates, lipids, nucleic acids) on their surface, forming a layer known as the biocorona. This process can change the performance of NPs in regard to pharmacokinetics and –dynamics, ranging from alterations in biodistribution and metabolism to toxicity and immune response.^[^ ^253^ ^]^ Recent findings demonstrated that the protein corona can also adversely affect the catalytic activity of nanozymes. For instance, nanorods with CAT, POD, and OXD activities, faced inhibitory effects from the blood protein corona, which hindered substrate permeation by blocking the active centers on their surface.^[^ ^254^ ^]^ In contrast, studies on MOF‐based nanocarriers revealed that these NPs can undergo significant biotransformation without affecting their safety and efficacy.^[^ ^255^, ^256^ ^]^ Neuer et al. have shown that nanoMOF‐based radio‐enhancers were effective in sarcoma cells and did not induce long‐term cytotoxicity despite their partial dissolution.^[^ ^255^ ^]^ These findings are encouraging and indicate a potential for inert NP dissolution, enabling stress‐free cellular clearance of degradation products.

After the uptake and initial interaction of NPs with the biological environment, NPs can be systemically distributed in the body and accumulate in different organs and tissues. The vast majority of the current in vivo studies (Table 3) observed nanozyme distribution to various organs including the liver, kidney, lung, brain, heart, and spleen. However, in most cases, NP accumulation in these tissues was minimal and toxicity was mostly absent. Elimination of nanozymes (if addressed) was mostly taking place through renal clearance,^[^ ^41^, ^46^, ^179^, ^181^, ^195^, ^223^ ^]^ with the exception of a nanozyme‐based liver fibrosis therapy study,^[^ ^191^ ^]^ where nanozyme elimination occurred through fecal matter. Although current evidence suggests efficient NP elimination, contributing to the reduction of undesired toxicity, further studies are warranted to exclude sublethal and long‐term effects.

### 6.4 Toxicological Considerations for Distinct Administration Routes

Depending on the intended administration route for the delivery of the nanozymes, different toxicity considerations should be taken into account (Figure 5).

Oral administration was implemented in a study focusing on novel antioxidant SAzymes coupled with probiotics for the treatment of inflammatory bowel disease.^[^ ^218^ ^]^ Administering a (nano)drug through the oral route to target GI disorders is ideal since it is the safest, most convenient, and effective way. Nevertheless, since the GI tract is one of the human biological barriers and thus a hostile environment (gut microbiota, acidic environment, digestive enzymes, and immune system patrolling) for foreign stimuli, it is necessary that the nanotherapeutic does not face biotransformation and degradation, which would result in undesirable efficacy decrease and toxicity. To this end, initially, nanomedicines should be challenged against simulation of digestive fluids and examine their durability.

Topical application of nanozymes was so far applied in two of the reviewed studies aiming for skin wound disinfection and healing.^[^ ^200^, ^257^ ^]^ For skin applications, it is necessary to consider that diseased skin or hair follicles could be putative entry routes for NPs into the deeper skin tissues (epidermis, dermis), lymph nodes, or even the blood stream.^[^ ^258^, ^259^, ^260^ ^]^ When the skin barrier has suffered mechanical or chemical breach, the penetration potential in deeper tissues is highly possible. In addition, the pH range of the stratum corneum is 5.4–5.9, which could lead to nanozyme dissolution and the release of toxic ions.^[^ ^258^ ^]^ Therefore, topical nanozyme treatment of (bacterial‐infected) skin wounds should ideally be coupled with a deeper‐tissue biodistribution assessment, pH‐driven nanozyme dissolution, and local toxicity assessment.

The SC administration route was also used for nanozyme‐based therapy of skin‐related disorders, namely diabetic ulcer wounds and wound abscesses.^[^ ^178^, ^204^ ^]^ With the SC administration, NPs are delivered in the cutis layer of skin, which host a small number of blood vessels and thus enabling a slow but continuous rate of absorption into the blood stream. Importantly, it should not be overlooked that nanomaterials present in skin layers below the stratum corneum have the capacity to induce or exacerbate allergic reactions, since it has been revealed that they could possess sensitization properties.^[^ ^239^, ^261^, ^262^ ^]^ Therefore, it is highly recommended that the sensitization potential of nanozymes delivered via the skin is carefully addressed to confirm their safety profile.

The IP administration route was utilized for the treatment of hyperglycemia and acute lung injury with nanozymes.^[^ ^213^, ^226^ ^]^ Therapeutics administered intraperitoneally are rapidly absorbed, but undergo first‐pass metabolism in the liver. While IP administration allows higher local concentrations with extended half‐life in the targeted organ, these advantages can be hampered by the possible local systemic toxicity.^[^ ^263^ ^]^ Notably, the pharmacological relevance of the IP route should be carefully considered, since it is rarely used in the clinical setting, primarily for peritoneal tumor therapy.^[^ ^264^, ^265^ ^]^

The most prevalent administration route was IV application, which was used for a variety of disease treatments, with cancer therapy being the leading one. The IV route provides controlled release, maximum bioavailability and fast activity onset of NPs. Nevertheless, the NPs have to overcome some obstacles, such as the mononuclear phagocytic system (MPS), which patrols and recognizes the opsonins attached on the NP surface, and renal clearance, were NPs are filtered out and further eliminated from the body. They are accounted, among others, for significant delivery insufficiency of a nanodrug to the diseased target organ.^[^ ^266^ ^]^ Therefore, uptake and effects in cells/tissues of the RES should be covered in the toxicity assessment of IV‐delivered therapeutic nanozymes even if surface coating strategies are employed to reduce opsonin binding.^[^ ^182^, ^266^ ^]^

### 6.5 Nanozyme Grouping Approach for Predictive Toxicity

From the evaluated cohort of studies, it is obvious that there are countless opportunities for nanozyme designs with a wealth of distinct physicochemical properties, thus requiring an individual toxicity evaluation of each NP. This is a general challenge in NP and chemical hazard assessment, which has stimulated researchers in the recent past to explore read‐across and grouping methods aiming for data gap filling and to estimate health hazards of NPs based on similarities in their main characteristics, such as size or chemical composition (Figure 5).^[^ ^267^, ^268^ ^]^ Such approaches could eventually be adopted for the targeted development of safe therapeutic nanozymes, especially since nanozymes should not just be considered as cargo‐packaged nanocarriers but also as a cargo themselves, due to their inherent catalytic activities. It would enable the prediction of undesirable biological responses, based also on the enzymatic activity among the other physicochemical characteristics.

### 6.6 Sex‐Specific Toxicity Responses

Despite the widely accepted physiological differences between males and females and the acknowledgment that sex is a decisive biological variable in biomedical and experimental research since the last century, this crucial variable has been neglected by the biomedical community and only recently has started to be considered (Figure 5).^[^ ^269^ ^]^ The gravity of this variable was heavily proven when US FDA during 1997–2000 withdrew eight prescription drugs from the market, due to higher female‐oriented adverse effects.^[^ ^269^ ^]^

Since the pharmacodynamics and ‐kinetics of a (nano)drug are tightly linked to the individual's physiology it is expected that sex‐specific parameters will exhibit notable shifts between males and females. For instance, a study performed in 2013 evaluated the potential sex‐dependent toxic effects of PEG‐coated Au NPs in mice. It was revealed that male mice suffered more significant liver damage, whereas female mice instead showed more kidney damage.^[^ ^270^ ^]^ Apart from obvious sex‐specific toxicity, metabolomics and proteomics data from male and female blood plasma demonstrated dramatic variations in the concentration and abundance of plasma components.^[^ ^271^ ^]^ Additionally, it has been repeatedly shown that plasma composition is prone to disease development‐dependent changes. As a result, blood plasma is a susceptible factor to various individual‐based physiological variables (e.g., sex, health status, age), which in turn influences the formation of NP‐biocorona and consequently the NP uptake pattern and intracellular fate.^[^ ^271^ ^]^

Further sex‐specific mechanisms, that can negatively impact the therapeutic performance and biosafety of nanomedicines, consist of molecular/cellular assemblies, such as X chromosome inactivation, sex‐specific immunity, and hormones, where even minor shifts can highly affect the physiology of a tissue and increase its susceptibility. Interestingly, disease environments are highly impacted by sex‐specific parameters as well, causing different therapeutic efficacy results.^[^ ^271^ ^]^ For instance, NP‐encapsulated MitoQ, a mitochondrium‐targeted antioxidant could be used as a therapeutic approach to alleviate placental oxidative stress, however, its efficiency was more pronounced in female placentae.^[^ ^272^ ^]^

Even though in the current cohort, the sex variability was not taken into account, it is encouraging that the vast majority of the studies disclose the strain, sex, and age of the animals in the in vivo studies, which could allow future comparability across studies.

Given the accumulating evidence, that sex has a remarkable impact on physiology and, as consequence, on NP‐mediated cellular responses, it is imperative to consider this biological variable to increase the robustness and relevance of results in future nanozyme‐based therapies.

### 6.7 Opportunities of Novel Omics‐ and Single Cell‐Based Approaches

While conventional approaches for the evaluation of NP‐cell interactions can provide useful data about the NP‐induced cellular effects, bulk analysis of cells hampers the identification of spatiotemporal‐ and cell heterogeneity‐induced responses. Moreover, traditional low throughput assays cannot reveal different cellular NP distribution patterns and complex molecular interactions and crosstalk. Hence, the traditional hazard and safety assessment of nanomedicines bears the risk of overlooking the cell/tissue heterogeneity aspect, generating convoluted NP‐cell interactions.

To this end, omics approaches in combination with single‐cell analysis enable an exhaustive understanding of subcellular events and mechanisms on different molecular levels (genome, epigenome, transcriptome, proteome), at single‐cell level that can also capture single‐cell identities and cell‐to‐cell molecular variations (Figure 5).^[^ ^273^, ^274^ ^]^ This could grant the development of cell‐specific disease treatment and monitoring of patient‐specific responses to certain therapies.^[^ ^273^ ^]^ A case in point is the very well‐studied, ubiquitous, and generally weighed as safe TiO_2_ and SiO_2_ NPs, which were shown, to alter the proteome and metabolome in lung and liver cells, respectively.^[^ ^275^, ^276^ ^]^

As conclusion, nanomedicine, and by extension nanozyme‐based therapies, could benefit from such novel approaches using human‐relevant advanced models, especially in the nanosafety assessment, since current conventional approaches fail to untangle the complex interactive network of molecular biomarkers.

### 6.8 The Importance of Personalized Medicine

Personalized medicine, an emerging and promising field of medicine, considers the unique genetic profile of patients in order to deliver patient‐tailored decisions regarding prevention, diagnosis, and treatment of disease (Figure 5).^[^ ^277^ ^]^ Given the multiple advantages that nanomedicine offers over conventional drugs, it is expected that it can decisively contribute to the advancement of personalized medicine. For instance, personalized therapeutic solutions can benefit from targeted nanocarriers for drug delivery purposes. Cancer has significantly triggered the development of personalized chemotherapeutics (e.g., imatinib^[^ ^278^ ^]^). Similarly, genome‐editing therapies have been advanced through personalized medicine approaches in the recent years for the treatment of genetic diseases.^[^ ^279^ ^]^ Nevertheless, in both cases, effective drug delivery is a crucial underlying factor for efficacy improvement and, in parallel, minimization of off‐target toxicity and immunogenicity.^[^ ^245^ ^]^ Body of evidence demonstrates that the formation of a biocorona on the NP surface is the decisive feature for organ distribution and tissue uptake.^[^ ^280^, ^281^, ^282^ ^]^ Thus, researchers have begun to focus on strategies for harnessing the biocorona to indirectly guide nanomedicines toward targeted biodistribution. This involves employing various surface coatings and functionalities.^[^ ^283^ ^]^

In the case of nanozymes, the situation becomes slightly more complex, since these particles serve a dual function: they act both as nanocarriers and as active therapeutic agents through their inherent catalytic activities. Besides improving targeting capabilities of nanozymes, to reach the organ/tissue in question, several challenges related to the catalytic performance need to be addressed. The lack of structural and morphological resemblance between conventional nanozymes and natural enzymes such as heterogeneous active sites and absence of fine structure (impact on enzymatic activity) as well as metal leakage (nanosafety aspect), led to the development of next‐generation SAzymes with well‐defined active catalytic sites that mimic the active center of natural enzymes. This advancement increased the substrate affinity, specificity, and catalytic activity, leading to a more targeted catalysis along with less metal release and potential side effects. Therefore, SAzymes can constitute the foundation of personalized next generation therapeutic nanozymes. Up to now, the SAzyme research field has set a strong focus on oxidoreductase activities due to the high demand for novel therapies to treat cancer and inflammatory diseases. Here, precision nanomedicine can be further harnessed to investigate patient‐specific intracellular oxidant/antioxidant enzyme balance, different ROS types and ratios, and damage to biomolecules.^[^ ^284^, ^285^, ^286^, ^287^ ^]^ This would enable a possible patient‐specific enzymatic activity‐dependent SAzyme synthesis in order to target the specific and dominant ROS damage. Nevertheless, more in‐depth multi‐disciplinary research efforts are warranted to untangle this complex field and provide future advanced patient‐specific nanozyme‐based therapies.

### 6.9 Closing Remarks

Here, we have summarized and discussed the current safety knowledge of therapeutic nanozymes. Despite different chemical composition and enzymatic activities, most nanozymes, conventional or modern SAzymes, showed no or low hazard potential. However, toxicity assessment mostly included a limited set of classical in vitro and in vivo endpoints that were addressed in simple 2D monoculture and mouse models, respectively. While animal data are associated with considerable uncertainties regarding their predictive value for humans, static 2D cell cultures grown on rigid plastic surfaces can fail to appropriately capture toxicity responses in physiological tissues.^[^ ^288^, ^289^, ^290^ ^]^ In the nanotoxicology field, the past decade has experienced the development of exciting advanced in vitro and ex vivo tissue models based on novel technologies including organoid cultures, microphysiological platforms, or 3D bioprinting that could be adopted in nanomedicine to achieve an improved human‐based hazard assessment and reduce the need for experimental animals.^[^ ^290^, ^291^ ^]^ In fact, the reciprocal knowledge transfer between these two sub‐disciplines, which evolved separately but share many common goals, is not yet leveraged to its full potential but is expected to drive the establishment of safe nanotechnologies.^[^ ^292^ ^]^ Therefore, we advocate that developers of therapeutic nanozymes should team up with nanotoxicologists to effectively accelerate the next‐generation nanozymes.

## Conflict of Interest

The authors declare no conflict of interest.

## Author Contributions

N.T. performed the investigation; visualized the idea for the study; wrote the original draft; and wrote, reviewed, and edited the final manuscript. H.S. wrote the original draft and wrote, reviewed, and edited the final manuscript. S.S. visualized the idea for the study; wrote the original draft; and wrote, reviewed, and edited the final manuscript. W.T. performed supervision; wrote, reviewed, and edited the final manuscript; and performed funding acquisition. Z.M. performed supervision; wrote, reviewed, and edited the final manuscript; and performed funding acquisition. T.B.‐T. conceptualized the idea for the study; performed supervision; wrote the original draft; visualized the idea for the study; wrote, reviewed, and edited the final manuscript; and performed the funding acquisition.
